# Identification of five genetic variants as novel determinants of type 2 diabetes mellitus in Japanese by exome-wide association studies

**DOI:** 10.18632/oncotarget.19287

**Published:** 2017-07-17

**Authors:** Yoshiji Yamada, Jun Sakuma, Ichiro Takeuchi, Yoshiki Yasukochi, Kimihiko Kato, Mitsutoshi Oguri, Tetsuo Fujimaki, Hideki Horibe, Masaaki Muramatsu, Motoji Sawabe, Yoshinori Fujiwara, Yu Taniguchi, Shuichi Obuchi, Hisashi Kawai, Shoji Shinkai, Seijiro Mori, Tomio Arai, Masashi Tanaka

**Affiliations:** ^1^ Department of Human Functional Genomics, Advanced Science Research Promotion Center, Mie University, Tsu, Japan; ^2^ CREST, Japan Science and Technology Agency, Kawaguchi, Japan; ^3^ Computer Science Department, College of Information Science, University of Tsukuba, Tsukuba, Japan; ^4^ RIKEN Center for Advanced Intelligence Project, Tokyo, Japan; ^5^ Department of Computer Science, Nagoya Institute of Technology, Nagoya, Japan; ^6^ Department of Internal Medicine, Meitoh Hospital, Nagoya, Japan; ^7^ Department of Cardiology, Kasugai Municipal Hospital, Kasugai, Japan; ^8^ Department of Cardiovascular Medicine, Inabe General Hospital, Inabe, Japan; ^9^ Department of Cardiovascular Medicine, Gifu Prefectural Tajimi Hospital, Tajimi, Japan; ^10^ Department of Molecular Epidemiology, Medical Research Institute, Tokyo Medical and Dental University, Tokyo, Japan; ^11^ Section of Molecular Pathology, Graduate School of Health Care Sciences, Tokyo Medical and Dental University, Tokyo, Japan; ^12^ Research Team for Social Participation and Community Health, Tokyo Metropolitan Institute of Gerontology, Tokyo, Japan; ^13^ Research Team for Promoting Support System for Home Care, Tokyo Metropolitan Institute of Gerontology, Tokyo, Japan; ^14^ Research Team for Social Participation and Health Promotion, Tokyo Metropolitan Institute of Gerontology, Tokyo, Japan; ^15^ Center for Promotion of Clinical Investigation, Tokyo Metropolitan Geriatric Hospital, Tokyo, Japan; ^16^ Department of Pathology, Tokyo Metropolitan Geriatric Hospital, Tokyo, Japan; ^17^ Department of Clinical Laboratory, Tokyo Metropolitan Geriatric Hospital, Tokyo, Japan

**Keywords:** diabetes mellitus, fasting plasma glucose, blood glycosylated hemoglobin, polymorphism, exome-wide association study

## Abstract

We performed exome-wide association studies to identify single nucleotide polymorphisms that either influence fasting plasma glucose level or blood hemoglobin A_1c_ content or confer susceptibility to type 2 diabetes mellitus in Japanese. Exome-wide association studies were performed with the use of Illumina Human Exome-12 DNA Analysis or Infinium Exome-24 BeadChip arrays and with 11,729 or 8635 subjects for fasting plasma glucose level or blood hemoglobin A_1c_ content, respectively, or with 14,023 subjects for type 2 diabetes mellitus (3573 cases, 10,450 controls). The relation of genotypes of 41,265 polymorphisms to fasting plasma glucose level or blood hemoglobin A_1c_ content was examined by linear regression analysis. After Bonferroni's correction, 41 and 17 polymorphisms were significantly (*P* < 1.21 × 10^−6^) associated with fasting plasma glucose level or blood hemoglobin A_1c_ content, respectively, with two polymorphisms (rs139421991, rs189305583) being associated with both. Examination of the relation of allele frequencies to type 2 diabetes mellitus with Fisher's exact test revealed that 87 polymorphisms were significantly (*P* < 1.21 × 10^−6^) associated with type 2 diabetes mellitus. Subsequent multivariable logistic regression analysis with adjustment for age and sex showed that four polymorphisms (rs138313632, rs76974938, rs139012426, rs147317864) were significantly (*P* < 1.44 × 10^−4^) associated with type 2 diabetes mellitus, with rs138313632 and rs139012426 also being associated with fasting plasma glucose and rs76974938 with blood hemoglobin A_1c_. Five polymorphisms—rs139421991 of *CAT*, rs189305583 of *PDCL2*, rs138313632 of *RUFY1*, rs139012426 of *LOC100505549*, and rs76974938 of *C21orf59*—may be novel determinants of type 2 diabetes mellitus.

## INTRODUCTION

Type 2 diabetes mellitus (DM) is a major cause of nephropathy, retinopathy, and neuropathy as well as cardiovascular disease and stroke [1, 2]. The heritability of type 2 DM has been estimated to be 50% to 60% [3]. Genome-wide association studies (GWASs) and meta-analyses thereof have identified >80 susceptibility loci for type 2 DM in individuals of European [4–9] or African [10] ancestry, in East Asians [11], or in multiple ethnic groups [12]. Genetic variants identified in these previous studies typically have a minor allele frequency (MAF) of ≥5% and a small individual effect size. Given that these common variants explain only a fraction of the heritability of type 2 DM, low-frequency (0.5% ≤ MAF < 5%) or rare (MAF < 0.5%) variants with larger effect sizes are also thought to contribute to the genetic architecture of this condition [13]. Among Japanese, GWASs have identified *KCNQ1* [14, 15], *UBE2E2*, *C2CD4A-B* [16], *ANK1* [17], *MIR129-LEP*, *GPSM1*, and *SLC16A13* [18] as susceptibility genes for type 2 DM, and a recent meta-analysis identified an additional seven susceptibility loci [19]. Genetic variants, including low-frequency and rare variants, that influence fasting plasma glucose (FPG) levels and blood glycosylated hemoglobin (HbA_1c_) content or which contribute to predisposition to type 2 DM in Japanese remain to be identified definitively, however.

We have now performed exome-wide association studies (EWASs) with the use of exome array–based genotyping methods to identify single nucleotide polymorphisms (SNPs)—especially low-frequency or rare coding variants with moderate to large effect sizes—that influence FPG levels and blood HbA_1c_ content or which confer susceptibility to type 2 DM in Japanese. We used Illumina arrays that provide coverage of functional SNPs in entire exons including low-frequency and rare variants.

## RESULTS

### Characteristics of subjects

The characteristics of subjects are shown in Table 1. Age, the frequency of men, body mass index, and the prevalence of hypertension, dyslipidemia, chronic kidney disease, and hyperuricemia as well as systolic and diastolic blood pressure, serum concentrations of triglycerides, creatinine, and uric acid, FPG level, and blood HbA_1c_ content were greater, whereas estimated glomerular filtration rate and the serum concentration of high density lipoprotein (HDL)–cholesterol were lower, in subjects with type 2 DM than in controls.

**Table 1:** Characteristics of the 14,023 study subjects

Characteristic	Type 2 DM	Controls	*P*
No. of subjects	3573	10,450	
Age (years)	66.2 ± 11.5	59.5 ± 13.8	<0.0001
Sex (male/female, %)	66.8/33.2	52.7/47.3	<0.0001
Body mass index (kg/m^2^)	24.2 ± 3.8	23.0 ± 3.3	<0.0001
Current or former smoker (%)	38.5	35.8	0.0067
Hypertension (%)	76.9	45.6	<0.0001
Systolic blood pressure (mmHg)	141 ± 26	128 ± 22	<0.0001
Diastolic blood pressure (mmHg)	78 ± 15	76 ± 13	<0.0001
Dyslipidemia (%)	75.4	56.7	<0.0001
Serum triglycerides (mmol/L)	1.68 ± 1.25	1.38 ± 0.92	<0.0001
Serum HDL-cholesterol (mmol/L)	1.31 ± 0.41	1.57 ± 0.46	<0.0001
Serum LDL-cholesterol (mmol/L)	3.08 ± 0.97	3.13 ± 0.81	0.0159
Chronic kidney disease (%)	34.5	20.4	<0.0001
Serum creatinine (μmol/L)	94.9 ± 123.5	73.1 ± 64.4	<0.0001
Estimated GFR (mL min^−1^ 1.73 m^−2^)	68.8 ± 24.1	73.4 ± 19.1	<0.0001
Hyperuricemia (%)	22.4	15.8	<0.0001
Serum uric acid (μmol/L)	336 ± 101	325 ± 92	<0.0001
Fasting plasma glucose (mmol/L)	8.81 ± 3.66	5.38 ± 0.77	<0.0001
Blood HbA_1c_ (%)	7.15 ± 1.73	5.48 ± 0.39	<0.0001

Quantitative data are means ± SD and were compared between subjects with type 2 DM and controls with the unpaired Student's *t* test. Categorical data were compared between the two groups with Fisher's exact test. Based on Bonferroni's correction, a *P* value of <0.0028 (0.05/18) was considered statistically significant. Hypertension: systolic blood pressure of ≥140 mmHg, diastolic blood pressure of ≥90 mmHg, or taking of anti-hypertensive medication. Dyslipidemia: serum triglyceride concentration of ≥1.65 mmol/L, serum high density lipoprotein (HDL)–cholesterol concentration of <1.04 mmol/L, serum low density lipoprotein (LDL)–cholesterol concentration of ≥3.64 mmol/L, or taking of anti-dyslipidemic medication. Chronic kidney disease: estimated glomerular filtration rate (eGFR) of <60 mL min^−1^ 1.73 m^−2^, where eGFR (mL min^−1^ 1.73 m^−2^) = 194 × (age in years)^−0.287^ × (serum creatinine in mg/dL)^−1.094^ (× 0.739 if female). Hyperuricemia: serum concentration of uric acid of >416 μmol/L.

### EWAS for FPG concentration

We examined the relation of genotypes of 41,265 SNPs that passed quality control to FPG levels in 11,729 subjects by linear regression analysis. A Manhattan plot for the EWAS is shown in Supplementary Figure 1A. After Bonferroni's correction, 41 SNPs were significantly [*P* < 1.21 × 10^−6^ (0.05/41,265)] associated with FPG concentration (Table 2).

**Table 2:** The 41 SNPs significantly (*P* < 1.21 × 10^−^^6^) associated with FPG level in the EWAS

Gene	dbSNP	Nucleotide (amino acid) substitution^a^	Chromosome: position	MAF (%)	*P* (genotype)
*OR4F6*	rs141569282	G/A (A117T)	15: 101806068	1.7	1.07 × 10^−12^
*SLC35F3*	rs140011243	T/G (C144G)	1: 234231563	0.5	1.56 × 10^−11^
*RUFY1*	rs138313632	T/G (S705A)	5: 179609505	0.5	9.91 × 10^−11^
*KARS*	rs201151665	A/T (M29L)	16: 75647967	0.4	3.98 × 10^−10^
*IFITM5*	rs146230729	G/T (P31T)	11: 299400	0.5	6.30 × 10^−10^
*CADM1*	rs561567580	G/C (R7P)	11: 115504375	0.4	6.76 × 10^−10^
*PPP1R9B*	rs113281588	G/C (G311A)	17: 50149582	0.5	1.57 × 10^−9^
*MUC17*	rs78010183	A/T (T1305S)	7: 101035329	1.8	1.63 × 10^−9^
*CCDC166*	rs75368383	T/C (K187R)	8: 143707454	0.5	1.70 × 10^−9^
*SCAMP4*	rs150715312	A/G (K69E)	19: 1912252	0.6	1.74 × 10^−9^
*PLEC*	rs201654895	A/G (M3874V)	8: 143918282	0.4	2.21 × 10^−9^
*LGR5*	rs117324318	G/A	12: 71440257	0.4	2.68 × 10^−9^
*CCDC114*	rs140189114	C/T (G632R)	19: 48297095	0.4	3.82 × 10^−9^
*NLRC3*	rs116433328	G/C (M286I)	16: 3564079	0.4	6.74 × 10^−9^
*CECR2*	rs201989565	G/A	22: 17548704	0.4	1.12 × 10^−8^
*DUS2*	rs202069030	G/C (R51S)	16: 68023050	0.4	1.14 × 10^−8^
*SIGLEC1*	rs201950990	A/C (V69G)	20: 3706550	0.5	2.11 × 10^−8^
*ALKBH1*	rs200168197	A/C (V329G)	14: 77673996	0.4	2.72 × 10^−8^
*TNFRSF4*	rs150516264	A/G (L98P)	1: 1213069	0.4	2.95 × 10^−8^
*LOC100505549*	rs139012426	G/C (S1242T)	18: 57648519	0.4	3.64 × 10^−8^
*ADAD2*	rs149894736	C/T (P107L)	16: 84191550	0.7	3.70 × 10^−8^
*YBEY*	rs200145138	C/G (L148V)	21: 46297572	0.4	3.84 × 10^−8^
*PRKCDBP*	rs11544766	C/G (T68S)	11: 6320274	0.4	5.38 × 10^−8^
*MYLIP*	rs201021082	T/C (V17A)	6: 16129372	0.4	5.47 × 10^−8^
*MGAT3*	rs201417286	T/G (V200G)	22: 39487946	0.3	6.54 × 10^−8^
*B3GNT6*	rs559157215	A/G (H301R)	11: 77040453	0.3	7.38 × 10^−8^
*KNDC1*	rs146093427	A/G (N546D)	10: 133195723	0.4	8.57 × 10^−8^
*TNXB*	rs141190850	T/C (D677G)	6: 32095823	0.3	8.62 × 10^−8^
*PCDHAC1*	rs185216314	A/G (Q479R)	5: 140968202	0.1	1.40 × 10^−7^
*CSPG4*	rs137981794	T/C (D1936G)	15: 75676712	0.4	1.50 × 10^−7^
*DNAJB2*	rs148615702	C/G (Q235E)	2: 219284715	0.3	1.63 × 10^−7^
*FGD3*	rs116496123	G/T	9: 93034624	0.7	1.71× 10^−7^
*AK8*	rs150636539	G/A (P328S)	9: 132792773	0.3	2.04 × 10^−7^
*LAMB3*	rs202068754	A/C (V753G)	1: 209623605	0.4	2.06 × 10^−7^
*GDPD3*	rs200801803	G/A	16: 30112132	0.3	2.95 × 10^−7^
*CAT*	rs139421991	G/A (R320Q)	11: 34456720	0.3	3.01 × 10^−7^
*OAS3*	rs62623451	G/A (A49T)	12: 112938675	0.3	3.13 × 10^−7^
*NOTCH1*	rs201053795	T/C (T970A)	9: 136509794	0.6	3.43 × 10^−7^
*PDCL2*	rs189305583	C/T (V69I)	4: 55580834	0.1	4.24 × 10^−7^
*SYNM*	rs200549249	G/A (G235E)	15: 99105903	0.2	7.54 × 10^−7^
*SNTB1*	rs145615160	A/G (Y57H)	8: 120811675	0.5	1.09 × 10^−6^

The relation of genotypes of SNPs to FPG level was examined by linear regression analysis. ^a^Major allele/minor allele.

### EWAS for blood HbA_1c_ content

We examined the relation of genotypes of 41,265 SNPs to blood HbA_1c_ content in 8635 subjects by linear regression analysis. A Manhattan plot for the EWAS is shown in Supplementary Figure 1B. After Bonferroni's correction, 17 SNPs were significantly (*P* < 1.21 × 10^−6^) associated with blood HbA_1c_ content (Table 3). SNPs rs139421991 [G/A (R320Q)] of *CAT* and rs189305583 [C/T (V69I)] of *PDCL2* were significantly associated with both FPG level and blood HbA_1c_ content.

**Table 3:** The 17 SNPs significantly (*P* < 1.21 × 10^−^^6^) associated with blood HbA_1c_ content in the EWAS

Gene	dbSNP	Nucleotide (amino acid) substitution^a^	Chromosome: position	MAF (%)	*P* (genotype)
*PTCHD3*	rs77473776	T/G (Q186K)	10: 27413695	30.6	3.59 × 10^−38^
*C21orf59*	rs76974938	C/T (D67N)	21: 32609946	2.4	1.68 × 10^−27^
*TNC*	rs138406927	C/T (A1096T)	9: 115064848	2.1	4.60 × 10^−27^
*KRR1*	rs17115182	G/A (P43S)	12: 75508405	7	5.17 × 10^−22^
*ZNF43*	rs149604219	G/A (A93V)	19: 21809741	3	7.87 × 10^−17^
*PRCP*	rs2229437	T/G (E133D)	11: 82853252	6.4	9.82 × 10^−12^
*CYP4F12*	rs609636	G/A (D76N)	19: 15678288	2.3	4.74 × 10^−11^
	rs1917321	A/C	11: 49356208	35.5	3.33 × 10^−10^
*LGALS14*	rs72480733	G/A (R27H)	19: 39705987	36.7	2.19 × 10^−8^
*ANKRD26*	rs12572862	C/G (L1219V)	10: 27033374	21.7	1.35 × 10^−7^
*CAT*	rs139421991	G/A (R320Q)	11: 34456720	0.3	1.69 × 10^−7^
*ZKSCAN3*	rs13201752	A/G (K200E)	6: 28363350	36.5	2.60 × 10^−7^
	rs10451497	C/T	19: 15093055	31.2	5.29 × 10^−7^
*OR8H3*	rs61751933	C/T (T16M)	11: 56122419	18.3	7.54 × 10^−7^
*PDCL2*	rs189305583	C/T (V69I)	4: 55580834	0.1	8.56 × 10^−7^
*HIVEP1*	rs200286173	A/G (Y374C)	6: 12120916	0.2	9.93 × 10^−7^
*RSL24D1*	rs200023487	A/G (V28A)	15: 55192832	0.1	1.20 × 10^−6^

The relation of genotypes of SNPs to blood HbA_1c_ content was examined by linear regression analysis. ^a^Major allele/minor allele.

### EWAS for type 2 DM

The EWAS for type 2 DM was performed with 14,023 subjects (3573 individuals with type 2 DM, 10,450 controls). We examined the relation of allele frequencies of 41,265 SNPs to type 2 DM with Fisher's exact test. A Manhattan plot for the EWAS is shown in Supplementary Figure 1C. After Bonferroni's correction, 87 SNPs were significantly (*P* < 1.21 × 10^−6^) associated with type 2 DM (Supplementary Table 1). The genotype distributions of these SNPs were in Hardy-Weinberg equilibrium (*P* > 0.001) among controls (Supplementary Table 2).

The relation of these 87 SNPs to type 2 DM was examined further by multivariable logistic regression analysis with adjustment for age and sex (Supplementary Table 3). Four SNPs—rs138313632 [T/G (S705A)] of *RUFY1*, rs76974938 [C/T (D67N)] of *C21orf59*, rs139012426 [G/C (S1242T)] of *LOC100505549*, and rs147317864 [C/T (A262T)] of *TRABD2B*—were significantly [*P* < 1.44 × 10^−4^ (0.05/348)] associated with type 2 DM (Table 4). The minor *G*, *T*, and *C* alleles of rs138313632, rs76974938, and rs139012426, respectively, were protective against type 2 DM, whereas the minor *T* allele of rs147317864 was a risk factor for this condition. SNPs rs138313632 of *RUFY1* and rs139012426 of *LOC100505549* were significantly associated with both FPG level and type 2 DM, whereas rs76974938 of *C21orf59* was significantly associated with both blood HbA_1c_ content and type 2 DM.

**Table 4:** Relation of SNPs to type 2 DM as determined by multivariable logistic regression analysis

Gene	SNP		Dominant		Recessive		Additive 1		Additive 2	
			*P*	OR (95% CI)	*P*	OR (95% CI)	*P*	OR (95% CI)	*P*	OR (95% CI)
*RUFY1*	rs138313632	T/G (S705A)	**4.20 × 10^−8^**	0.20 (0.09–0.39)	ND		**4.20 × 10^−8^**	0.20 (0.09–0.39)	ND	
*C21orf59*	rs76974938	C/T (D67N)	**1.00 × 10^−23^**	0.33 (0.26–0.41)	ND		**1.00 × 10^−23^**	0.33 (0.26–0.41)	ND	
*LOC100505549*	rs139012426	G/C (S1242T)	**2.28 × 10^−6^**	0.22 (0.09–0.44)	ND		**2.28 × 10^−6^**	0.22 (0.09–0.44)	ND	
*TRABD2B*	rs147317864	C/T (A262T)	**2.25 × 10^−5^**	1.21 × 10^8^ (ND)	ND		**2.25 × 10^−5^**	1.21 × 10^8^ (ND)	ND	

Multivariable logistic regression analysis was performed with adjustment for age and sex. Based on Bonferroni's correction, *P* values of <1.44 × 10^−4^ (0.05/348) were considered statistically significant and are shown in bold. OR, odds ratio; CI, confidence interval; ND, not determined.

### Relation of SNPs to FPG level or blood HbA_1c_ content

We examined the relation of genotypes of identified SNPs to the FPG level or blood HbA_1c_ content by one-way analysis of variance (ANOVA). The 41 SNPs identified in the EWAS for FPG level, including the two SNPs (rs138313632, rs139012426) also found to be associated with type 2 DM, were all significantly [*P* < 0.0012 (0.05/43)] associated with FPG level. The remaining two SNPs associated with type 2 DM (rs76974938, rs147317864) were not significantly related to the FPG level (Table 5).

**Table 5:** Relation of SNPs to FPG level

SNP		FPG (mmol/L)		*P*
Associated with FPG level and type 2 DM				
rs138313632	T/G (S705A)	*TT*	*TG*	
		6.25 ± 2.45	4.92 ± 1.66	**9.91 × 10^−11^**
rs139012426	G/C (S1242T)	*GG*	*GC*	
		6.24 ± 2.44	4.96 ± 1.83	**3.64 × 10^−8^**
Associated with FPG level				
rs141569282	G/A (A117T)	*GG*	*GA*	
		6.38 ± 2.65	5.50 ± 0.90	**1.07 × 10^−12^**
rs140011243	T/G (C144G)	*TT*	*TG*	
		6.24 ± 2.44	4.87 ± 1.63	**1.56 × 10^−11^**
rs201151665	A/T (M29L)	*AA*	*AT*	
		6.24 ± 2.44	4.81 ± 1.14	**3.98 × 10^−10^**
rs146230729	G/T (P31T)	*GG*	*GT*	
		6.24 ± 2.44	4.98 ± 1.72	**6.30 × 10^−10^**
rs561567580	G/C (R7P)	*GG*	*GC*	
		6.24 ± 2.44	4.96 ± 1.74	**6.76 × 10^−10^**
rs113281588	G/C (G311A)	*GG*	*GC*	
		6.22 ± 2.44	5.00 ± 1.73	**1.57 × 10^−9^**
rs78010183	A/T (T1305S)	*AA*	*AT*	
		6.25 ± 2.47	5.63 ± 1.39	**1.63 × 10^−9^**
rs75368383	T/C (K187R)	*TT*	*TC*	
		6.25 ± 2.44	5.02 ± 1.69	**1.70 × 10^−9^**
rs150715312	A/G (K69E)	*AA*	*AG*	
		6.25 ± 2.45	5.15 ± 1.70	**1.74 × 10^−9^**
rs201654895	A/G (M3874V)	*AA*	*AG*	
		6.24 ± 2.44	4.96 ± 1.19	**2.21 × 10^−9^**
rs117324318	G/A	*GG*	*GA*	
		6.24 ± 2.44	5.01 ± 1.73	**2.68 × 10^−9^**
rs140189114	C/T (G632R)	*CC*	*CT*	
		6.24 ± 2.44	4.96 ± 1.75	**3.82 × 10^−9^**
rs116433328	G/C (M286I)	*GG*	*GC*	
		6.24 ± 2.44	4.93 ± 1.77	**6.74 × 10^−9^**
rs201989565	G/A	*GG*	*GA*	
		6.24 ± 2.44	5.00 ± 1.76	**1.12 × 10^−8^**
rs202069030	G/C (R51S)	*GG*	*GC*	
		6.24 ± 2.44	4.91 ± 1.74	**1.14 × 10^−8^**
rs201950990	A/C (V69G)	*AA*	*AC*	
		6.24 ± 2.44	5.08 ± 1.79	**2.11 × 10^−8^**
rs200168197	A/C (V329G)	*AA*	*AC*	
		6.24 ± 2.44	4.97 ± 1.84	**2.72 × 10^−8^**
rs150516264	A/G (L98P)	*AA*	*AG*	
		6.24 ± 2.44	5.03 ± 1.81	**2.95 × 10^−8^**
rs149894736	C/T (P107L)	*CC*	*CT*	
		6.24 ± 2.44	5.25 ± 1.57	**3.70 × 10^−8^**
rs200145138	C/G (L148V)	*CC*	*CG*	
		6.24 ± 2.44	4.98 ± 1.92	**3.84 × 10^−8^**
rs11544766	C/G (T68S)	*CC*	*CG*	
		6.24 ± 2.44	5.01 ± 1.77	**5.38 × 10^−8^**
rs201021082	T/C (V17A)	*TT*	*TC*	
		6.24 ± 2.43	4.98 ± 1.89	**5.47 × 10^−8^**
rs201417286	T/G (V200G)	*TT*	*TG*	
		6.24 ± 2.44	4.98 ± 1.85	**6.54 × 10^−8^**
rs559157215	A/G (H301R)	*AA*	*AG*	
		6.24 ± 2.43	4.90 ± 1.87	**7.38 × 10^−8^**
rs146093427	A/G (N546D)	*AA*	*AG*	
		6.24 ± 2.44	5.09 ± 1.80	**8.57 × 10^−8^**
rs141190850	T/C (D677G)	*TT*	*TC*	
		6.24 ± 2.43	4.91 ± 1.92	**8.62 × 10^−8^**
rs185216314	A/G (Q479R)	*AA*	*AG*	
		6.21 ± 2.41	8.38 ± 6.21	**1.40 × 10^−7^**
rs137981794	T/C (D1936G)	*TT*	*TC*	
		6.24 ± 2.44	5.06 ± 1.88	**1.50 × 10^−7^**
rs148615702	C/G (Q235E)	*CC*	*CG*	
		6.24 ± 2.44	4.86 ± 2.00	**1.63 × 10^−7^**
rs116496123	G/T	*GG*	*GT*	
		6.24 ± 2.46	5.36 ± 1.87	**1.71 × 10^−7^**
rs150636539	G/A (P328S)	*GG*	*GA*	
		6.24 ± 2.44	4.93 ± 1.88	**2.04 × 10^−7^**
rs202068754	A/C (V753G)	*AA*	*AC*	
		6.24 ± 2.44	5.10 ± 1.86	**2.06 × 10^−7^**
rs200801803	G/A	*GG*	*GA*	
		6.24 ± 2.44	4.88 ± 1.98	**2.95 × 10^−7^**
rs139421991	G/A (R320Q)	*GG*	*GA*	
		6.17 ± 2.35	7.43 ± 3.08	**3.01 × 10^−7^**
rs62623451	G/A (A49T)	*GG*	*GA*	
		6.24 ± 2.44	5.03 ± 1.85	**3.13 × 10^−7^**
rs201053795	T/C (T970A)	*TT*	*TC*	
		6.24 ± 2.45	5.27 ± 1.87	**3.43 × 10^−7^**
rs189305583	C/T (V69I)	*CC*	*CT*	
		6.21 ± 2.41	8.26 ± 5.49	**4.24 × 10^−7^**
rs200549249	G/A (G235E)	*GG*	*GA*	
		6.23 ± 2.44	4.76 ± 1.12	**7.54 × 10^−7^**
rs145615160	A/G (Y57H)	*AA*	*AG*	
		6.24 ± 2.44	5.26 ± 2.15	**1.09 × 10^−6^**
Associated with type 2 DM				
rs76974938	C/T (D67N)	*CC*	*CT*	
		6.21 ± 2.46	6.46 ± 2.27	0.0758
rs147317864	C/T (A262T)	*CC*		
		6.21 ± 2.46		ND

Data were compared among genotypes by one-way ANOVA. Based on Bonferroni's correction, *P* values of <0.0012 (0.05/43) were considered statistically significant and are shown in bold. ND, not determined.

The 17 SNPs identified in the EWAS for blood HbA_1c_ content, including the one SNP (rs76974938) also found to be associated with type 2 DM, were all significantly [*P* < 0.0025 (0.05/20)] associated with blood HbA_1c_ content by one-way ANOVA. The remaining three SNPs associated with type 2 DM (rs138313632, rs139012426, rs147317864) were not significantly related to blood HbA_1c_ content (Table 6).

**Table 6:** Relation of SNPs to blood HbA_1c_ content

SNP			Blood HbA_1c_ (%)		*P*
Associated with blood HbA_1c_ content and type 2 DM					
rs76974938	C/T (D67N)	*CC*	*CT*		
		6.04 ± 1.25	5.47 ± 0.68		**<1.0 × 10^−23^**
Associated with blood HbA_1c_ content					
rs77473776	T/G (Q186K)	*TT*	*TG*	*GG*	
		6.03 ± 1.28	5.85 ± 1.12	5.47 ± 0.63	**<1.0 × 10^−23^**
rs138406927	C/T (A1096T)	*CC*	*CT*		
		6.04 ± 1.25	5.44 ± 0.61		**<1.0 × 10^−23^**
rs17115182	G/A (P43S)	*GG*	*GA*		
		6.04 ± 1.25	5.48 ± 0.57		**5.20 × 10^−22^**
rs149604219	G/A (A93V)	*GG*	*GA*		
		5.95 ± 1.21	5.44 ± 0.51		**7.87 × 10^−17^**
rs2229437	T/G (E133D)	*TT*	*TG*	*GG*	
		5.89 ± 1.14	6.15 ± 1.44	6.26 ± 1.32	**6.94 × 10^−11^**
rs609636	G/A (D76N)	*GG*	*GA*		
		6.01 ± 1.24	5.31 ± 0.50		**4.74 × 10^−11^**
rs1917321	A/C	*AA*	*AC*	*CC*	
		5.87 ± 1.09	6.02 ± 1.26	6.10 ± 1.37	**1.11 × 10^−9^**
rs72480733	G/A (R27H)	*GG*	*GA*	*AA*	
		5.85 ± 1.10	5.94 ± 1.23	6.09 ± 1.30	**9.51 × 10^−8^**
rs12572862	C/G (L1219V)	*CC*	*CG*	*GG*	
		5.92 ± 1.21	6.06 ± 1.33	6.18 ± 1.34	**8.99 × 10^−7^**
rs139421991	G/A (R320Q)	*GG*	*GA*		
		5.89 ± 1.14	6.64 ± 1.50		**1.69 × 10^−7^**
rs13201752	A/G (K200E)	*AA*	*AG*	*GG*	
		6.05 ± 1.24	5.90 ± 1.20	5.87 ± 1.09	**3.47 × 10^−7^**
rs10451497	C/T	*CC*	*CT*	*TT*	
		6.02 ± 1.21	5.93 ± 1.27	5.78 ± 0.92	**2.03 × 10^−6^**
rs61751933	C/T (T16M)	*CC*	*CT*	*TT*	
		6.04 ± 1.26	5.88 ± 1.16	5.91 ± 1.04	**8.58 × 10^−7^**
rs189305583	C/T (V69I)	*CC*	*CT*		
		5.90 ± 1.17	7.00 ± 2.87		**8.56 × 10^−7^**
rs200286173	A/G (Y374C)	*AA*	*AG*		
		5.90 ± 1.17	6.99 ± 2.46		**9.93 × 10^−7^**
rs200023487	A/G (V28A)	*AA*	*AG*		
		5.90 ± 1.17	7.04 ± 2.55		**1.20 × 10^−6^**
Associated with type 2 DM					
rs138313632	T/G (S705A)	*TT*			
		5.90 ± 1.18			ND
rs139012426	G/C (S1242T)	*GG*	*GC*		
		5.90 ± 1.18	8.2		0.0504
rs147317864	C/T (A262T)	*CC*			
		6.02 ± 1.24			ND

Data were compared among genotypes by one-way ANOVA. Based on Bonferroni's correction, *P* values of <0.0025 (0.05/20) were considered statistically significant and are shown in bold. ND, not determined.

### Relation of identified SNPs to phenotypes examined in previous GWASs

We examined the genes, chromosomal loci, and SNPs identified in the present study to DM-related phenotypes examined in previous GWASs deposited in a public database [GWAS Catalog (http://www.ebi.ac.uk/gwas)]. *LGR5* has been previously shown to be related to type 2 DM, whereas *TNXB* has been previously associated with type 1 DM and *PTCHD3* with fasting insulin-related traits (Supplementary Table 4). The remaining 54 SNPs identified in the present study have not been previously found to be related to DM-related phenotypes.

### Network analysis of the genes identified in the present study

We performed network analysis of the top ten (high scores) genes that have been shown to be associated with type 2 DM selected from DisGeNET database (http://www.disgenet.org/web/DisGeNET) and four genes (*CAT*, *PDCL2*, *RUFY1*, and *C21orf59*) identified in the present study by the use of Cytoscape version 3.4.0 software (http://www.cytoscape.org/). Given that *LOC100505549* protein has not been characterized, it could not be examined. The network analysis showed that *CAT*, *PDCL2*, *RUFY1*, and *C21orf59* have potential indirect interactions with several genes previously shown to be associated with type 2 DM (Supplementary Figure 2).

## DISCUSSION

We have now shown that two SNPs—rs139421991 [G/A (R320Q)] of *CAT* and rs189305583 [C/T (V69I)] of *PDCL2*—were significantly associated with both FPG levels and blood HbA_1c_ content; two SNPs—rs138313632 [T/G (S705A)] of *RUFY1* and rs139012426 [G/C (S1242T)] of *LOC100505549*—were significantly associated with both FPG levels and type 2 DM; and one SNP, rs76974938 [C/T (D67N)] of *C21orf59*, was significantly associated with both blood HbA_1c_ content and type 2 DM. These five SNPs may thus be novel determinants of type 2 DM. Given that FPG levels are affected by meals of the day before examination, we selected the SNPs and genes that were associated with both FPG levels and blood HbA_1c_ or type 2 DM.

The catalase gene (*CAT*) is located at chromosomal region 11p13 (NCBI Gene, https://www.ncbi.nlm.nih.gov/gene) and is expressed in various tissues and organs including the pancreas (The Human Protein Atlas, http://www.proteinatlas.org). Catalase catalyzes the breakdown of hydrogen peroxide into oxygen and water. Inherited catalase deficiency has been associated with an increased prevalence of type 2 DM in Hungarians [20–22]. The frequency of various *CAT* mutations has been found to be increased in individuals with DM, especially in females with type 2 DM, and such inherited catalase deficiency is associated with an early onset of type 2 DM [20]. We have now shown that rs139421991 [G/A (R320Q)] of *CAT* was significantly associated with both FPG levels and blood HbA_1c_ content, with the minor *A* allele being related to an increase in these parameters. This association of *CAT* with type 2 DM may be attributable to the role of the encoded protein in the metabolism of hydrogen peroxide and oxidative stress, although the underlying molecular mechanism remains to be determined.

The phosducin like 2 gene (*PDCL2*) is located at chromosome 4q12 (NCBI Gene) and is expressed at a high level in testis (The Human Protein Atlas). The PDCL2 protein belongs to the phosducin family [23]. PDCL1 has been shown to be essential for G protein signaling as a result of its role in folding and assembly of the Gβγ dimer. PDCL2 and PDCL3 likely assist in the folding of actin, tubulin, and proteins that activate cell cycle progression [24]. We have now shown that rs189305583 [C/T (V69I)] of *PDCL2* was significantly associated with both the FPG concentration and blood HbA_1c_ content, with the minor *T* allele being related to increases in these parameters. Given that G protein–coupled receptor signaling promotes insulin secretion and the proliferation of pancreatic β cells [25], the association *PDCL2* with FPG levels and blood HbA_1c_ content may be attributable to an effect of PDCL2 on such signaling, although the molecular mechanism remains unclear.

The RUN and FYVE domain containing 1 gene (*RUFY1*) is located at chromosomal region 5q35.3 (NCBI Gene) and is expressed in various tissues and organs including the pancreas (The Human Protein Atlas). The RUFY1 protein binds to phosphatidylinositol 3-phosphate and promotes early endosomal trafficking including the tethering and fusion of vesicles through interactions with small GTPases such as Rab4, Rab5, and Rab14 [26]. We have now shown that rs138313632 [T/G (S705A)] of *RUFY1* was significantly associated with both FPG concentration and type 2 DM, with the minor *G* allele being related to a decreased FPG level and a reduced risk for type 2 DM. Given that small GTPases enhances insulin granule exocytosis [27], the association of *RUFY1* with both FPG levels and type 2 DM may be attributable to an effect of the encoded protein on insulin secretion.

The uncharacterized *LOC100505549* gene is located at chromosome 18q21.31 (NCBI Gene). The function of *LOC100505549* remains unknown. We have now shown that rs139012426 [G/C (S1242T)] of *LOC100505549* was significantly associated with both FPG levels and type 2 DM, with the minor *C* allele being related to a decreased FPG concentration and a reduced risk of type 2 DM, although the functional relevance of this association remains unknown.

The chromosome 21 open reading frame 59 gene (*C21orf59*) is located at chromosome 21q22.11 (NCBI Gene) and is expressed in various tissues and organs including the pancreas (The Human Protein Atlas). The C21orf59 protein promotes dynein arm assembly in motile cilia, and mutations in *C21orf59* cause ciliary dyskinesia [28]. Ciliopathies are associated with pancreatic defects that manifest mostly as cysts originating from ductal cells. Ciliary proteins have been suggested to influence insulin secretion and energy regulation [29, 30]. We have now shown that rs76974938 [C/T (D67N)] of *C21orf59* was significantly associated with both blood HbA_1c_ content and type 2 DM, with the minor *T* allele being related to a decreased blood HbA_1c_ content and a reduced risk for type 2 DM. Given that *C21orf59* may activate ciliary function and that cilia influence insulin secretion [29, 30], the association of *C21orf59* with blood HbA_1c_ content and type 2 DM might reflect an effect of this gene on insulin secretion, although the underlying molecular mechanism remains unclear.

In previous GWASs of type 2 DM in the Japanese population [14–19], the MAF of identified SNPs ranged from 2% to 48% and the odds ratio (OR) from 0.38 to 1.70. In a meta-analysis of GWASs for type 2 DM in East Asian populations [11] and in a trans-ancestry meta-analysis of GWASs for type 2 DM [12], the OR ranged from 1.06 to 1.10 or from 1.08 to 1.13, respectively. In our study, we identified four SNPs associated with type 2 DM, with the MAF and OR in a dominant model of logistic regression analysis for rs138313632, rs76974938, rs139012426, and rs147317864 being 0.5% and 0.20, 2.4% and 0.33, 0.4% and 0.22, and 0.2% and 1.21 × 10^8^, respectively. Both rs138313632 and rs76974938 were thus low-frequency variants with a moderate effect size, whereas rs139012426 and rs147317864 were rare variants with a moderate to large effect size.

Two SNPs (MAF and differences in FPG level or blood HbA_1c_ content between genotypes, respectively, shown in parentheses) associated with both FPG concentration and blood HbA_1c_ content—rs139421991 of *CAT* (0.3%, 17.0%, 11.3%) and rs189305583 of *PDCL2* (0.1%, 24.8%, 15.7%)—were rare variants with a large effect size; two SNPs associated with both FPG levels and type 2 DM—rs138313632 of *RUFY1* (0.5%, 21.3%, 22.4%) and rs139012426 of *LOC100505549* (0.4%, 20.5%, 28.0%)—were rare or low-frequency variants with a large effect size; and one SNP associated with both blood HbA_1c_ content and type 2 DM, rs76974938 of *C21orf59* (2.4%, 4.3%, 9.4%), was a low-frequency variant with a moderate effect size. Among the remaining 37 SNPs associated with FPG levels, 25 SNPs were rare variants with a large effect size and 12 SNPs were low-frequency variants with a moderate to large effect size. Among the remaining 14 SNPs associated with blood HbA_1c_ content, two SNPs were rare variants with a large effect size, three SNPs were low-frequency variants with a moderate to large effect size, and nine SNPs were common variants with a small to moderate effect size (Supplementary Table 5).

There are several limitations to the present study. (i) Given that our results were not replicated, they will require validation in other subject panels or in other ethnic groups. (ii) There is a possibility that some control individuals are prediabetic. (iii) It is possible that SNPs identified in the present study are in linkage disequilibrium with other polymorphisms in other nearby genes that are actually determinants of FPG levels, blood HbA_1c_ content, or the development of type 2 DM. (iv) One SNP associated with type 2 DM was not significantly related to FPG level or blood HbA_1c_ content, a discrepancy that may be attributable to the effects of medical treatment. (v) The biological or functional evidence of the association of the identified SNPs with FPG level, blood HbA_1c_ content, or type 2 DM remains to be determined. Because of lack of experiments for functional analyses, the association of the SNPs identified in the present study with type 2 DM, FPG levels, or blood HbA_1c_ content should be interpreted carefully.

In conclusion, we have identified five SNPs—rs139421991 [G/A (R320Q)] of *CAT*, rs189305583 [C/T (V69I)] of *PDCL2*, rs138313632 [T/G (S705A)] of *RUFY1*, rs139012426 [G/C (S1242T)] of LOC100505549, and rs76974938 [C/T (D67N)] of *C21orf59*—as novel determinants of type 2 DM. We also identified 37, 14, or one SNPs as candidate determinants of FPG levels, blood HbA_1c_ content, and type 2 DM, respectively. Determination of genotypes for these SNPs may prove informative for assessment of the genetic risk for type 2 DM in Japanese.

## MATERIALS AND METHODS

### Study subjects

A total of 14,023 individuals was examined. The subjects were recruited as described previously [31].

Type 2 DM was defined according to the criteria of the World Health Organization as described previously [32–34]. Subjects with type 2 DM had an FPG level of ≥6.93 mmol/L (126 mg/dL) or a blood HbA_1c_ content of ≥6.5% or were taking antidiabetes medication. We thus examined 3573 subjects with type 2 DM and 10,450 controls. Individuals with type 1 DM, maturity-onset diabetes of the young, DM associated with mitochondrial diseases or single-gene disorders, pancreatic diseases, or other metabolic or endocrinologic diseases were excluded from the study. Those taking medications that may cause secondary DM were also excluded. The control subjects had an FPG level of <6.05 mmol/L (110 mg/dL), a blood HbA_1c_ content of <6.2%, and no history of DM or of having taken antidiabetes medication. Autopsy cases were excluded from controls.

The study protocol complied with the Declaration of Helsinki and was approved by the Committees on the Ethics of Human Research of Mie University Graduate School of Medicine, Hirosaki University Graduate School of Medicine, Tokyo Metropolitan Institute of Gerontology, and participating hospitals. Written informed consent was obtained from all subjects or families of the deceased subjects.

### EWASs

Methods for sample collection and extraction of genomic DNA have been described previously [31]. EWASs for FPG concentration and blood HbA_1c_ content included 11,729 and 8635 subjects, respectively, whereas that for type 2 DM included 14,023 individuals (3573 subjects with type 2 DM, 10,450 controls). Data for FPG levels were obtained from subjects who had fasted overnight. Data for blood HbA_1c_ content were obtained from subjects with type 2 DM or impaired glucose tolerance or from those who had annual health checkup. The EWASs were performed with the use of a HumanExome-12 v1.1 or v1.2 DNA Analysis BeadChip or Infinium Exome-24 v1.0 BeadChip (Illumina, San Diego, CA, USA). Detailed information of the exome arrays and methods of quality control have been described previously [31]. Genotype data were examined for population stratification by principal components analysis [35] (Supplementary Figure 3). A total of 41,265 SNPs passed quality control and was subjected to analysis.

### Statistical analysis

The relation of genotypes of SNPs to FPG level or blood HbA_1c_ content in the EWASs was examined by linear regression analysis. For analysis of characteristics of the study subjects, quantitative and categorical data were compared between individuals with type 2 DM and controls with the unpaired Student's *t* test or Fisher's exact test, respectively. Allele frequencies were estimated by the gene counting method, and Fisher's exact test was used to identify departure from Hardy-Weinberg equilibrium. The relation of allele frequencies of SNPs to type 2 DM in the EWAS was examined with Fisher's exact test. To compensate for multiple comparisons of genotypes with FPG level or blood HbA_1c_ content or of allele frequencies with type 2 DM, we applied Bonferroni's correction for statistical significance of association. Given that 41,265 SNPs were analyzed, the significance level was set at *P* < 1.21 × 10^−6^ (0.05/41,265) for the EWASs. Quantile-quantile plots for *P* values of genotypes in the EWASs for FPG level or blood HbA_1c_ content or for those of allele frequencies in the EWAS for type 2 DM are shown in Supplementary Figure 4. The inflation factor (λ) was 1.02 for FPG level, 1.03 for blood HbA_1c_ content, and 1.26 for type 2 DM. Multivariable logistic regression analysis was performed with type 2 DM as a dependent variable and independent variables including age, sex (0, woman; 1, man), and genotype of each SNP. A detailed method of analysis has been described previously [31]. The relation of genotypes of identified SNPs to FPG level or blood HbA_1c_ content was examined by one-way ANOVA. Bonferroni's correction was also applied to other statistical analysis as indicated to compensate for multiple comparisons. Statistical tests were performed with JMP Genomics version 6.0 software (SAS Institute, Cary, NC, USA).

## SUPPLEMENTARY MATERIALS FIGURES AND TABLES




