# Supplementary material for: Identification of five genetic variants as novel determinants of type 2 diabetes mellitus in Japanese by exome-wide association studies
Source: Oncotarget. 2017 Jul 17;8(46):80492–505. doi: 10.18632/oncotarget.19287 (PMC5655215; doi:10.18632/oncotarget.19287)
Supplement: Supplementary file 1 [file oncotarget-08-80492-s001.pdf]

Identification of five genetic variants as novel determinants of type 2 diabetes mellitus in Japanese by exome-wide association studies

SUPPLEMENTARY MATERIALS

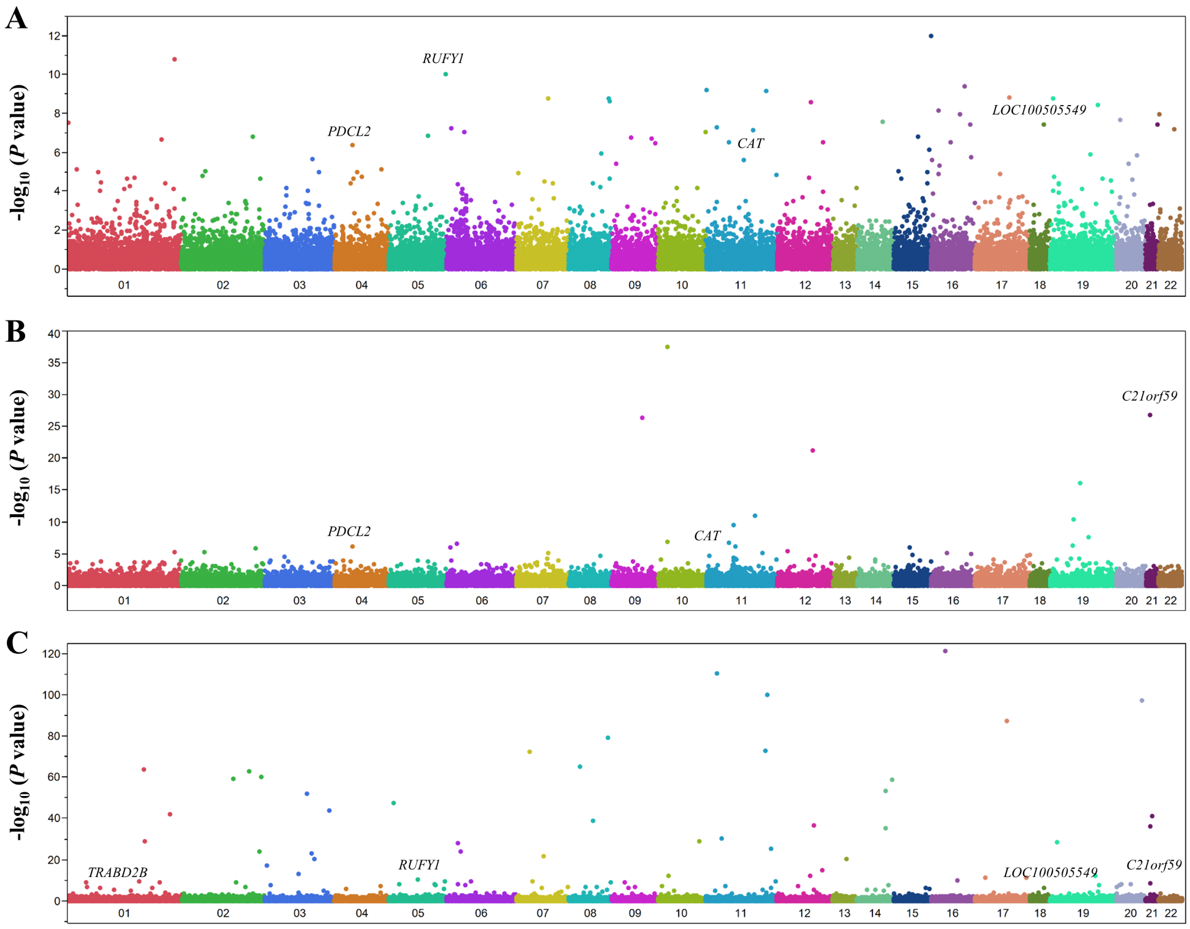

Supplementary Figure 1: Manhattan plots for *P* values in the EWASs of FPG level **(A)**, blood HbA_{1c} content **(B)**, and type 2 DM **(C).** The *P* values (*y*-axis) are plotted as $-\log_{10}(P)$ with respect to the physical chromosomal position of the corresponding SNPs (*x*-axis). The genes found to be associated with both FPG level and either blood HbA_{1c} content or type 2 DM are indicated in (A), those associated with both blood HbA_{1c} content and either FPG level or type 2 DM are indicated in (B), and those associated with type 2 DM are indicated in (C).

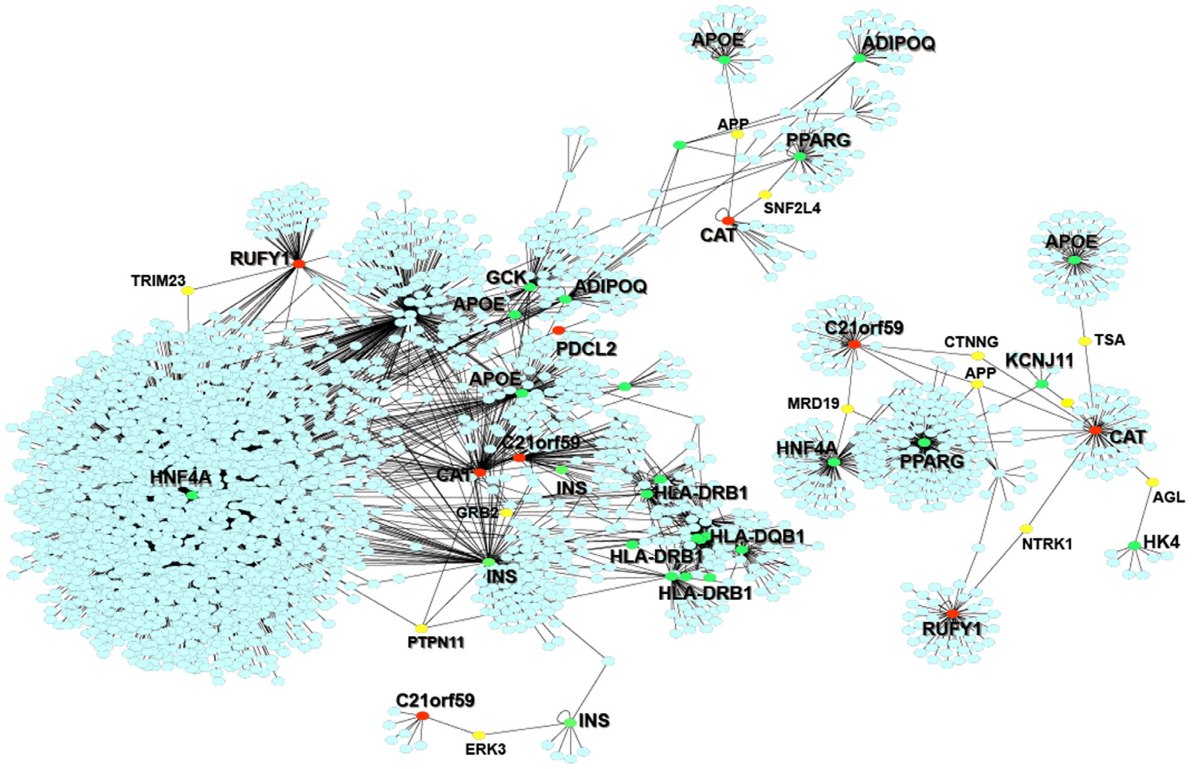

Supplementary Figure 2: The network of putative interactions among molecules related to the top ten (high scores) genes that have been shown to be associated with type 2 DM (closed green circle) selected from DisGeNET database (http://www.disgenet.org/web/ DisGeNET) and four genes (*CAT, PDCL2, RUFY1,* and *C21orf59*) identified in the present study (closed red circle) examined by Cytoscape version 3.4.0 software (http://www.cytoscape.org/). Molecules shown in closed yellow circle represent putative mediators of interactions between those genes. The network was automatically integrated based on human molecular interaction data from different public databases.

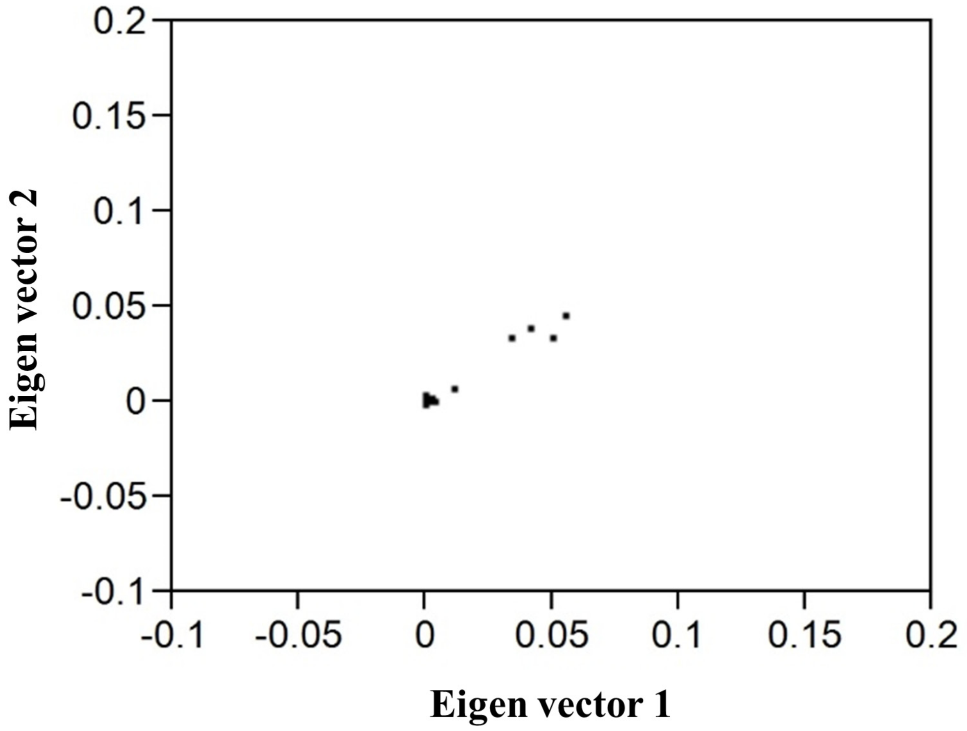

Supplementary Figure 3: Distribution of samples examined by principal components analysis for population stratification in the EWASs for type 2 DM. The analysis was performed with the EIGENSTRAT method, with the samples being plotted according to the first (horizontal axis) and second (vertical axis) principal components.

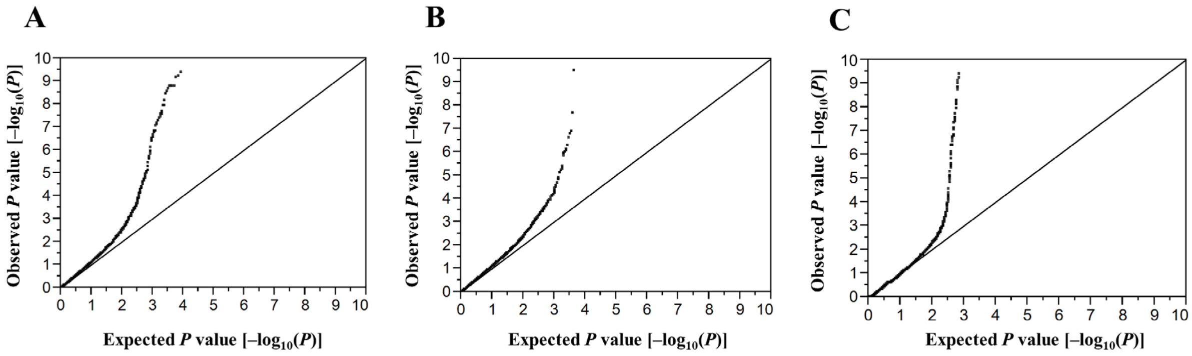

Supplementary Figure 4: Quantile-quantile plots for *P* values of genotypes in the EWASs for FPG level **(A)** or blood HbA_{1c} content **(B)** or for those of allele frequencies in the EWAS for type 2 DM **(C).** The observed *P* values (*y*-axis) were compared with the expected *P* values (*x*-axis) under the null hypothesis, with the values being plotted as $-\log_{10}(P)$.

Supplementary Table 1: The 87 SNPs significantly ($P < 1.21 \times 10^{-6}$) associated with type 2 DM in the EWAS

See Supplementary File 1

Supplementary Table 2: Genotype distributions for SNPs associated ($P < 1.21 \times 10^{-6}$) with type 2 DM in the EWAS

See Supplementary File 1

Supplementary Table 3: Relation of SNPs to type 2 DM as determined by multivariable logistic regression analysis

See Supplementary File 1

Supplementary Table 4: Relation of genes, chromosomal loci, and SNPs identified in the present study to phenotypes previously examined in GWASs

See Supplementary File 1

Supplementary Table 5: Minor allele frequencies and effect sizes of the 56 SNPs identified in the present study

See Supplementary File 1
