# Supplementary material for: Identification of five genetic variants as novel determinants of type 2 diabetes mellitus in Japanese by exome-wide association studies
Source: Oncotarget. 2017 Jul 17;8(46):80492–505. doi: 10.18632/oncotarget.19287 (PMC5655215; doi:10.18632/oncotarget.19287)
Supplement: Supplementary file 2 [file oncotarget-08-80492-s002.docx]

**Supplementary Table 1.** **The 87 SNPs significantly (*P* < 1.21 × 10^–6^) associated with type 2 DM in the EWAS**

________________________________________________________________________________

Gene dbSNP Nucleotide Chromosome: MAF *P* (allele) Allele

(amino acid) position (%) OR

substitution^a^

________________________________________________________________________________

*TMC5* rs150481868 A/G (N426S) 16: 19463816 2.1 3.72 × 10^–122^ 0.89

*DNHD1* rs2344828 G/A (R697Q) 11: 6528774 12.7 2.12 × 10^–111^ 0.95

*PHLDB1* rs145947849 G/A (R414Q) 11: 118628064 0.1 7.89 × 10^–101^ 0.52

*LSM14B* rs200803813 C/A (P176H) 20: 62129884 0.7 6.17 × 10^–98^ 0.94

*ARHGAP27* rs2959953 C/G (P213R) 17: 45429642 48.6 6.59 × 10^–88^ 0.97

*ZC3H3* rs150994390 C/T (R160Q) 8: 143538888 1.5 6.73 × 10^–80^ 0.88

*NXPE2* rs149918157 A/G (H370R) 11: 114705961 1.4 1.00 × 10^–73^ 1.12

*AOAH* rs2228410 C/T (D28N) 7: 36724067 48.8 4.99 × 10^–73^ 0.99

*CDCA2* rs116429520 T/C (I114T) 8: 25462162 0.3 4.30 × 10^–66^ 1.55

*FMO4* rs190463354 G/A (R223H) 1: 171332749 0.5 9.92 × 10^–65^ 0.95

*BARD1* rs2070094 C/T (V488M) 2: 214767531 36.5 1.13 × 10^–63^ 1.01

*ESPNL* rs184614603 G/A (R913Q) 2: 238131452 0.6 9.66 × 10^–61^ 1.12

*LRP2* rs143822500 T/C (T867A) 2: 169257164 0.7 6.54 × 10^–60^ 0.78

*C14orf180* rs150513093 C/T (R18C) 14: 104586482 0.9 2.17 × 10^–59^ 0.92

*NRDE2* rs117406130 T/C 14: 90331935 1.9 5.35 × 10^–54^ 1.03

*CD86* rs2681417 A/G (V185I) 3: 122106350 0.2 9.14 × 10^–53^ 1.00

*FBXL7* rs257747 C/A 5: 15817857 42.7 3.34 × 10^–48^ 1.00

*HRG* rs10770 T/C (I180T) 3: 186671770 9.5 9.09 × 10^–45^ 0.97

*PYCR2* rs201142342 T/C (I203V) 1: 225921578 0.4 1.03 × 10^–42^ 0.94

*TTC3* rs1053966 C/G (H1751D) 21: 37195708 17.8 7.64 × 10^–42^ 0.99

*CA3* rs20571 G/A (V31I) 8: 85439768 38.5 1.59 × 10^–39^ 0.94

*C12orf50* rs4454801 A/G 12: 88020411 5.8 3.82 × 10^–37^ 1.04

*URB1* rs200312105 C/T (V995M) 21: 32349333 0.6 7.19 × 10^–37^ 1.19

*RPS6KA5* rs8018102 G/A 14: 91023104 31.4 6.98 × 10^–36^ 1.01

*CTR9* rs75206030 C/G (P981R) 11: 10775263 0.2 7.91 × 10^–31^ 0.77

rs1419138 C/T 10: 117971770 42.2 1.64 × 10^–29^ 1.04

*PRRC2C* rs760644 A/G (T906A) 1: 171540188 9.7 2.10 × 10^–29^ 0.91

*DUS3L* rs200981144 C/T (E211K) 19: 5787093 0.2 5.52 × 10^–29^ 1.30

*MOG* rs29234 T/G 6: 29656335 0.8 6.15 × 10^–29^ 0.99

*OR6T1* rs150534954 C/G (C155W) 11: 123943374 1.4 3.65 × 10^–26^ 1.03

*PSORS1C1* rs3130977 T/C 6: 31114212 25.1 1.07 × 10^–24^ 0.98

rs12614237 T/G 2: 237280635 37.9 1.62 × 10^–24^ 0.96

*GATA2* rs78245253 G/C (A250P) 3: 128485850 4.6 9.36 × 10^–24^ 1.04

*PEX1* rs144825021 G/T (A723D) 7: 92494621 0.4 1.70 × 10^–22^ 0.95

*DIS3* rs144957541 G/A (S587F) 13: 72768908 0.3 4.33 × 10^–21^ 0.99

*AMOTL2* rs1353776 G/C (E729D) 3: 134358628 5.0 4.53 × 10^–21^ 1.07

*CPNE9* rs139476663 T/C (V87A) 3: 9704994 0.4 5.81 × 10^–18^ 0.77

*ATXN2* rs191400641 T/A (I762N) 12: 111509950 1.1 1.10 × 10^–15^ 1.08

*MINA* rs2172257 T/C (T385A) 3: 97945881 28.3 1.10 × 10^–13^ 0.98

rs11177192 T/C 12: 68370545 44.5 5.25 × 10^–13^ 1.03

LOC101929951 rs7081678 G/A 10: 31701695 2.6 5.31 × 10^–13^ 1.03

*EML2* rs12151009 T/C (M180V) 19: 45638587 9.0 5.46 × 10^–13^ 1.05

LOC101928766 rs1078485 A/G 17: 79920407 49.4 5.15 × 10^–12^ 0.97

*SHBG* rs13894 G/A (R126C) 17: 7626584 6.4 9.29 × 10^–12^ 0.98

rs4702982 C/T 5: 100655944 24.8 5.88 × 10^–11^ 1.03

rs11645831 G/A 16: 60207120 42.7 8.42 × 10^–11^ 0.98

*BRPF3* rs3748045 C/G 6: 36230800 31.0 4.04 × 10^–10^ 0.96

rs11977526 A/G 7: 45968511 21.8 4.20 × 10^–10^ 1.01

*LY9* rs509749 G/A (V512M) 1: 160823770 25.4 4.23 × 10^–10^ 1.03

*RUFY1* rs138313632 T/G (S705A) 5: 179609505 0.5 5.72 × 10^–10^ 0.17

rs2155378 G/A 11: 132404106 42.2 6.09 × 10^–10^ 1.05

*UBXN11* rs138559558 G/A (R289C) 1: 26284470 1.2 7.52 × 10^–10^ 0.88

*TJP2* rs144396411 G/A (A89T) 9: 69218351 1.2 1.01 × 10^–9^ 0.88

rs947211 G/A 1: 205783537 49.2 1.43 × 10^–9^ 0.98

*TTN* rs55675869 C/T (V33366I) 2: 178537013 6.0 1.56 × 10^–9^ 1.03

*OPLAH* rs7004867 C/T (R31Q) 8: 144059941 0.3 1.81 × 10^–9^ 1.38

*C21orf59* rs76974938 C/T (D67N) 21: 32609946 2.4 4.96 × 10^–9^ 0.52

rs967417 T/C 20: 6640246 13.8 5.57 × 10^–9^ 0.99

*HMGXB3* rs2241698 G/A (V263I) 5: 150010585 7.9 6.49 × 10^–9^ 1.06

rs1992660 G/A 5: 40414965 14.5 7.98 × 10^–9^ 0.94

rs892666 T/C 6: 29956951 25.0 8.15 × 10^–9^ 1.02

*EIF6* rs78127944 C/T (R661H) 20: 35286797 2.0 1.09 × 10^–8^ 0.91

*ADAM33* rs41453444 A/T (H612L) 20: 3671651 1.4 1.82 × 10^–8^ 1.09

*TULP2* rs34378208 C/T (G122S) 19: 48895148 0.2 1.84 × 10^–8^ 0.58

*SERPINA6* rs2228541 C/A (A246S) 14: 94309884 13.1 2.66 × 10^–8^ 0.95

*BTNL2* rs9268507 G/A 6: 32409762 30.2 3.14 × 10^–8^ 1.04

*IRGM* rs72553867 C/A (T94K) 5: 150848404 15.4 3.20 × 10^–8^ 1.05

*GRIP2* rs61731939 G/A (T833M) 3: 14505690 10.4 4.19 × 10^–8^ 1.04

rs6536541 T/C 4: 160846662 15.0 4.43 × 10^–8^ 1.10

*MEIG1* rs7919322 G/A 10: 14961279 41.3 4.58 × 10^–8^ 0.98

*SPATS2* rs191343457 C/G (P202R) 12: 49496911 0.2 8.08 × 10^–8^ 1.11

*PRMT3* rs10741838 T/C 11: 20425873 46.5 9.38 × 10^–8^ 1.01

*NBEAL1* rs2351524 C/T 2: 203016269 0.7 1.54 × 10^–7^ 1.48

*SLC25A32* rs141856398 T/C (Y300C) 8: 103400460 1.0 1.65 × 10^–7^ 0.93

*TRIB3* rs35051116 G/A (R153H) 20: 391453 0.1 1.85 × 10^–7^ 1.27

*ZNF683* rs10794531 C/T (R53H) 1: 26367754 37.7 1.90 × 10^–7^ 0.99

*LINC01492* rs10820412 T/C 9: 103296308 49.7 2.24 × 10^–7^ 1.01

*SPIDR* rs2306928 A/G 8: 47599185 0.4 2.67 × 10^–7^ 0.67

*LINC01006* rs849084 T/C 7: 156560335 48.1 2.69 × 10^–7^ 0.99

*KIF7* rs12906938 T/C 15: 89648124 33.9 3.60 × 10^–7^ 1.02

rs668853 A/G 9: 82696232 35.4 3.83 × 10^–7^ 1.00

*OR8D4* rs61907183 C/T (R122C) 11: 123906795 0.6 4.09 × 10^–7^ 1.01

*LOC100505549* rs139012426 G/C (S1242T) 18: 57648519 0.4 4.11 × 10^–7^ 0.20

rs7807771 A/G 7: 85519647 5.0 4.20 × 10^–7^ 0.92

rs12068912 T/C 1: 195311294 8.2 7.26 × 10^–7^ 1.09

*TRABD2B* rs147317864 C/T (A262T) 1: 47801502 0.2 7.35 × 10^–7^ ND

*ADAMTS17* rs4246302 A/G 15: 100147762 30.5 1.03 × 10^–6^ 1.01

________________________________________________________________________________

Allele frequencies were analyzed with the Fisher’s exact test. ^a^Major allele/minor allele. ND, not determined.

**Supplementary Table 2.** **Genotype distributions for SNPs associated (*P* < 1.21 × 10^–6^) with type 2 DM in the EWAS**

____________________________________________________________________________________________________________________

SNP Type 2 DM H-W *P* Controls H-W *P*

____________________________________________________________________________________________________________________

rs150481868 A/G (N426S) *AA* *AG* *GG* *AA* *AG* *GG*

3434 (96.11) 138 (3.86) 1 (0.03) 1.0000 9998 (95.67) 444 (4.25) 8 (0.08) 0.1700

rs2344828 G/A (R697Q) *GG* *GA* *AA*  *GG* *GA* *AA*

2754 (77.08) 753 (21.07) 66 (1.85) 0.0895 7904 (75.64) 2374 (22.72) 171 (1.64) 0.6654

rs145947849 G/A (R414Q) *GG* *GA* *AA*  *GG* *GA* *AA*

3568 (99.86) 5 (0.14) 0 (0) 1.0000 10422 (99.73) 28 (0.27) 0 (0) 1.0000

rs200803813 C/A (P176H) *CC* *CA* *AA*  *CC* *CA* *AA*

3520 (98.52) 53 (1.48) 0 (0) 1.0000 10285 (98.43) 164 (1.57) 0 (0) 1.0000

rs2959953 C/G (P213R) *CC* *CG* *GG*  *CC* *CG* *GG*

965 (27.14) 1765 (49.63) 826 (23.23) 0.7370 2746 (26.46) 5131 (49.44) 2501 (24.10) 0.2800

rs150994390 C/T (R160Q) *CC* *CT* *TT*  *CC* *CT* *TT*

3471 (97.31) 96 (2.69) 0 (0) 1.0000 10123 (96.97) 313 (3.00) 3 (0.03) 0.7371

rs149918157 A/G (H370R) *AA* *AG* *GG* *AA* *AG* *GG*

3467 (97.06) 103 (2.88) 2 (0.06) 0.1878 10167 (97.33) 278 (2.66) 1 (0.01) 1.0000

rs2228410 C/T (D28N) *CC* *CT* *TT*  *CC* *CT* *TT*

944 (26.44) 1783 (49.95) 843 (23.61) 1.0000 2756 (26.37) 5175 (49.52) 2519 (24.11) 0.3576

rs116429520 T/C (I114T) *TT* *TC* *CC*  *TT* *TC* *CC*

3547 (99.27) 26 (0.73) 0 (0) 1.0000 10401 (99.53) 49 (0.47) 0 (0) 1.0000

rs190463354 G/A (R223H) *GG* *GA* *AA*  *GG* *GA* *AA*

3541 (99.10) 32 (0.90) 0 (0) 1.0000 10351 (99.05) 99 (0.95) 0 (0) 1.0000

rs2070094 C/T (V488M) *CC* *CT* *TT*  *CC* *CT* *TT*

1437 (40.23) 1639 (45.88) 496 (13.89) 0.4083 4185 (40.05) 4861 (46.52) 1404 (13.43) 0.9162

rs184614603 G/A (R913Q) *GG* *GA* *AA*  *GG* *GA* *AA*

3513 (98.54) 52 (1.46) 0 (0) 1.0000 10286 (98.70) 136 (1.30) 0 (0) 1.0000

rs143822500 T/C (T867A) *TT* *TC* *CC*  *TT* *TC* *CC*

3533 (98.88) 40 (1.12) 0 (0) 1.0000 10301 (98.57) 148 (1.42) 1 (0.01) 0.4164

rs150513093 C/T (R18C) *CC* *CT* *TT*  *CC* *CT* *TT*

3514 (98.35) 59 (1.65) 0 (0) 1.0000 10263 (98.21) 187 (1.79) 0 (0) 1.0000

rs117406130 T/C *TT* *TC* *CC*  *TT* *TC* *CC*

3434 (96.11) 137 (3.83) 2 (0.06) 0.6487 10055 (96.22) 390 (3.73) 5 (0.05) 0.4363

rs2681417 A/G (V185I) *AA* *AG* *GG* *AA* *AG* *GG*

3557 (99.55) 16 (0.45) 0 (0) 1.0000 10403 (99.55) 47 (0.45) 0 (0) 1.0000

rs257747 C/A *CC* *CA* *AA*  *CC* *CA* *AA*

1167 (32.67) 1743 (48.80) 662 (18.53) 0.8111 3420 (32.73) 5088 (48.70) 1940 (18.57) 0.5359

rs10770 T/C (I180T) *TT* *TC* *CC*  *TT* *TC* *CC*

2945 (82.42) 597 (16.71) 31 (0.87) 0.9203 8572 (82.03) 1766 (16.90) 112 (1.07) 0.0534

rs201142342 T/C (I203V) *TT* *TC* *CC*  *TT* *TC* *CC*

3544 (99.19) 29 (0.81) 0 (0) 1.0000 10361 (99.15) 88 (0.84) 1 (0.01) 0.1751

rs1053966 C/G (H1751D) *CC* *CG* *GG*  *CC* *CG* *GG*

2415 (67.61) 1043 (29.20) 114 (3.19) 0.9089 7059 (67.56) 3035 (29.05) 354 (3.39) 0.2183

rs20571 G/A (V31I) *GG* *GA* *AA*  *GG* *GA* *AA*

1391 (38.93) 1680 (47.02) 502 (14.05) 0.9148 3925 (37.56) 4898 (46.88) 1626 (15.56) 0.1334

rs4454801 A/G *AA* *AG* *GG* *AA* *AG* *GG*

3160 (88.44) 400 (11.20) 13 (0.36) 0.8812 9276 (88.76) 1146 (10.97) 28 (0.27) 0.2780

rs200312105 C/T (V995M) *CC* *CT* *TT*  *CC* *CT* *TT*

3518 (98.65) 48 (1.35) 0 (0) 1.0000 10317 (98.87) 118 (1.13) 0 (0) 1.0000

rs8018102 G/A *GG* *GA* *AA*  *GG* *GA* *AA*

1670 (46.75) 1535 (42.97) 367 (10.28) 0.6158 4936 (47.24) 4444 (42.53) 1069 (10.23) 0.1406

rs75206030 C/G (P981R) *CC* *CG* *GG*  *CC* *CG* *GG*

3562 (99.69) 11 (0.31) 0 (0) 1.0000 10409 (99.61) 40 (0.38) 1 (0.01) 0.0404

rs1419138 C/T *CC* *CT* *TT*  *CC* *CT* *TT*

1175 (32.90) 1728 (48.39) 668 (18.71) 0.4732 3505 (33.54) 5126 (49.06) 1818 (17.40) 0.4573

rs760644 A/G (T906A) *AA* *AG* *GG* *AA* *AG* *GG*

2955 (82.70) 589 (16.49) 29 (0.81) 1.0000 8495 (81.30) 1855 (17.75) 99 (0.95) 0.9120

rs200981144 C/T (E211K) *CC* *CT* *TT*  *CC* *CT* *TT*

3554 (99.47) 18 (0.50) 1 (0.03) 0.0263 10405 (99.57) 45 (0.43) 0 (0) 1.0000

rs29234 T/G *TT* *TG* *GG*  *TT* *TG* *GG*

3523 (98.60) 49 (1.37) 1 (0.03) 0.1645 10301 (98.57) 147 (11.41) 2 (0.02) 0.1021

rs150534954 C/G (C155W) *CC* *CG* *GG*  *CC* *CG* *GG*

3018 (97.20) 87 (2.80) 0 (0) 1.0000 10171 (97.33) 273 (2.61) 6 (0.06) 0.0130

rs3130977 T/C *TT* *TC* *CC*  *TT* *TC* *CC*

2020 (56.54) 1335 (37.36) 218 (6.10) 0.9285 5866 (56.13) 3890 (37.23) 694 (6.64) 0.1541

rs12614237 T/G *TT* *TG* *GG*  *TT* *TG* *GG*

1391 (38.93) 1696 (47.47) 486 (13.60) 0.4109 4010 (38.37) 4894 (46.83) 1546 (14.80) 0.4073

rs78245253 G/C (A250P) *GG* *GC* *CC*  *GG* *GC* *CC*

3232 (90.46) 333 (9.32) 8 (0.22) 1.0000 9499 (90.90) 922 (8.82) 29 (0.28) 0.1880

rs144825021 G/T (A723D) *GG* *GT* *TT*  *GG* *GT* *TT*

3541 (99.13) 30 (0.84) 1 (0.03) 0.0674 10351 (99.06) 98 (0.94) 0 (0) 1.0000

rs144957541 G/A (S587F) *GG* *GA* *AA*  *GG* *GA* *AA*

3552 (99.44) 20 (0.56) 0 (0) 1.0000 10386 (99.44) 59 (0.56) 0 (0) 1.0000

rs1353776 G/C (E729D) *GG* *GC* *CC*  *GG* *GC* *CC*

3219 (90.09) 337 (9.43) 17 (0.48) 0.0169 9458 (90.51) 969 (9.27) 23 (0.22) 0.8322

rs139476663 T/C (V87A) *TT* *TC* *CC*  *TT* *TC* *CC*

3548 (99.30) 25 (0.70) 0 (0) 1.0000 10355 (99.09) 95 (0.91) 0 (0) 1.0000

rs191400641 T/A (I762N) *TT* *TA* *AA*  *TT* *TA* *AA*

3487 (97.59) 86 (2.41) 0 (0) 1.0000 10218 (97.79) 229 (2.19) 2 (0.02) 0.3720

rs2172257 T/C (T385A) *TT* *TC* *CC*  *TT* *TC* *CC*

1854 (51.92) 1438 (40.27) 279 (7.81) 1.0000 5345 (51.16) 4260 (40.77) 843 (8.07) 0.9044

rs11177192 T/C *TT* *TC* *CC*  *TT* *TC* *CC*

1064 (29.78) 1798 (50.32) 711 (19.90) 0.3441 3244 (31.04) 5168 (49.46) 2038 (19.50) 0.8119

rs7081678 G/A *GG* *GA* *AA*  *GG* *GA* *AA*

3379 (94.62) 191 (5.35) 1 (0.03) 0.5203 9905 (94.82) 532 (5.09) 9 (0.09) 0.4443

rs12151009 T/C (M180V) *TT* *TC* *CC*  *TT* *TC* *CC*

2928 (81.95) 615 (17.21) 30 (0.84) 0.7699 8633 (82.66) 1733 (16.58) 79 (0.76) 0.4756

rs1078485 A/G *AA* *AG* *GG* *AA* *AG* *GG*

944 (26.42) 1769 (49.51) 860 (24.07) 0.5922 2688 (25.73) 5161 (49.39) 2600 (24.88) 0.2177

rs13894 G/A (R126C) *GG* *GA* *AA*  *GG* *GA* *AA*

3136 (87.77) 421 (11.78) 16 (0.45) 0.5749 9152 (87.59) 1247 (11.93) 50 (0.48) 0.2909

rs4702982 C/T *CC* *CT* *TT*  *CC* *CT* *TT*

2027 (56.73) 1297 (36.30) 249 (6.97) 0.0366 5928 (56.74) 3915 (37.48) 604 (5.78) 0.2141

rs11645831 G/A *GG* *GA* *AA*  *GG* *GA* *AA*

1191 (33.33) 1737 (48.62) 645 (18.05) 0.7841 3416 (32.69) 5132 (49.11) 1902 (18.20) 0.7492

rs3748045 C/G *CC* *CG* *GG*  *CC* *CG* *GG*

1724 (48.25) 1523 (42.63) 326 (9.12) 0.7222 4938 (47.25) 4470 (42.78) 1042 (9.97) 0.5100

rs11977526 A/G *AA* *AG* *GG*  *AA* *AG* *GG*

2193 (61.38) 1199 (33.56) 181 (5.06) 0.3036 6402 (61.26) 3560 (34.07) 488 (4.67) 0.8403

rs509749 G/A (V512M) *GG* *GA* *AA*  *GG* *GA* *AA*

1942 (54.35) 1415 (39.60) 216 (6.05) 0.0496 5850 (55.98) 3924 (37.55) 676 (6.47) 0.6042

rs138313632 T/G (S705A) *TT* *TG* *GG*  *TT* *TG* *GG*

3490 (99.77) 8 (0.23) 0 (0) 1.0000 10107 (98.66) 137 (1.34) 0 (0) 1.0000

rs2155378 G/A *GG* *GA* *AA*  *GG* *GA* *AA*

1145 (32.06) 1794 (50.22) 633 (17.72) 0.1328 3563 (34.10) 5059 (48.42) 1827 (17.48) 0.6726

rs138559558 G/A (R289C) *GG* *GA* *AA*  *GG* *GA* *AA*

3494 (97.79) 79 (2.21) 0 (0) 1.0000 10191 (97.52) 257 (2.46) 2 (0.02) 0.6783

rs144396411 G/A (A89T) *GG* *GA* *AA*  *GG* *GA* *AA*

3497 (97.87) 76 (2.13) 0 (0) 1.0000 10199 (97.60) 250 (2.39) 1 (0.01) 1.0000

rs947211 G/A *GG* *GA* *AA*  *GG* *GA* *AA*

963 (26.95) 1728 (48.36) 882 (24.69) 0.0563 2758 (26.39) 5077 (48.59) 2615 (25.02) 0.0040

rs55675869 C/T (V33366I) *CC* *CT* *TT*  *CC* *CT* *TT*

3149 (88.13) 408 (11.42) 16 (0.45) 0.4676 9237 (88.39) 1174 (11.24) 39 (0.37) 0.7941

rs7004867 C/T (R31Q) *CC* *CT* *TT*  *CC* *CT* *TT*

3547 (99.27) 26 (0.73) 0 (0) 1.0000 10395 (99.47) 55 (0.53) 0 (0) 1.0000

rs76974938 C/T (D67N) *CC* *CT* *TT*  *CC* *CT* *TT*

2859 (97.15) 84 (2.85) 0 (0) 1.0000 8813 (94.60) 503 (5.40) 0 (0) 0.0022

rs967417 T/C *TT* *TC* *CC*  *TT* *TC* *CC*

2656 (74.34) 850 (23.79) 67 (1.87) 1.0000 7733 (74.03) 2530 (24.22) 183 (1.75) 0.1516

rs2241698 G/A (V263I) *GG* *GA* *AA*  *GG* *GA* *AA*

3012 (84.30) 540 (15.11) 21 (0.59) 0.6541 8916 (85.33) 1459 (13.96) 74 (0.71) 0.0977

rs1992660 G/A *GG* *GA* *AA*  *GG* *GA* *AA*

2645 (74.05) 852 (23.85) 75 (2.10) 0.4885 7600 (72.74) 2598 (24.86) 251 (2.40) 0.1039

rs892666 T/C *TT* *TC* *CC*  *TT* *TC* *CC*

1950 (55.16) 1374 (38.87) 211 (5.97) 0.1429 5868 (56.64) 3818 (36.85) 675 (6.51) 0.1095

rs78127944 C/T (R661H) *CC* *CT* *TT*  *CC* *CT* *TT*

3441 (96.31) 130 (3.64) 2 (0.05) 0.3560 10023 (95.91) 424 (4.06) 3 (0.03) 0.8050

rs41453444 A/T (H612L) *AA* *AT* *TT*  *AA* *AT* *TT*

3476 (97.29) 92 (2.57) 5 (0.14) 0.0007 10177 (97.39) 271 (2.59) 2 (0.02) 0.7021

rs34378208 C/T (G122S) *CC* *CT* *TT*  *CC* *CT* *TT*

3563 (99.72) 10 (0.28) 0 (0) 1.0000 10401 (99.53) 48 (0.46) 1 (0.01) 0.0571

rs2228541 C/A (A246S) *CC* *CA* *AA*  *CC* *CA* *AA*

2721 (76.16) 798 (22.33) 54 (1.51) 0.6506 7851 (75.13) 2425 (23.21) 174 (1.66) 0.4194

rs9268507 G/A *GG* *GA* *AA*  *GG* *GA* *AA*

1706 (47.76) 1540 (43.11) 326 (9.13) 0.4316 5127 (49.07) 4394 (42.05) 928 (8.88) 0.7795

rs72553867 C/A (T94K) *CC* *CA* *AA*  *CC* *CA* *AA*

2513 (70.35) 973 (27.24) 86 (2.41) 0.5340 7493 (71.78) 2686 (25.73) 260 (2.49) 0.2919

rs61731939 G/A (T833M) *GG* *GA* *AA*  *GG* *GA* *AA*

2477 (79.78) 591 (19.03) 37 (1.19) 0.7783 8414 (80.52) 1912 (18.30) 124 (1.18) 0.1871

rs6536541 T/C *TT* *TC* *CC*  *TT* *TC* *CC*

2525 (70.69) 952 (26.65) 95 (2.66) 0.6188 7595 (72.72) 2615 (25.04) 234 (2.24) 0.6132

rs7919322 G/A *GG* *GA* *AA*  *GG* *GA* *AA*

1247 (34.90) 1720 (48.14) 606 (16.96) 0.7558 3573 (34.19) 5072 (48.54) 1805 (17.27) 0.9518

rs191343457 C/G (P202R) *CC* *CG* *GG*  *CC* *CG* *GG*

3557 (99.55) 16 (0.45) 0 (0) 1.0000 10408 (99.60) 42 (0.40) 0 (0) 1.0000

rs10741838 T/C *TT* *TC* *CC*  *TT* *TC* *CC*

1005 (28.14) 1785 (49.99) 781 (21.87) 0.8403 2958 (28.31) 5218 (49.94) 2273 (21.75) 0.7682

rs2351524 C/T *CC* *CT* *TT*  *CC* *CT* *TT*

3503 (98.04) 70 (1.96) 0 (0) 1.0000 10313 (98.69) 135 (1.29) 2 (0.02) 0.0770

rs141856398 T/C (Y300C) *TT* *TC* *CC*  *TT* *TC* *CC*

3509 (98.21) 64 (1.79) 0 (0) 1.0000 10249 (98.08) 200 (1.91) 1 (0.01) 0.6250

rs35051116 G/A (R153H) *GG* *GA* *AA*  *GG* *GA* *AA*

3560 (99.64) 13 (0.36) 0 (0) 1.0000 10420 (99.71) 30 (0.29) 0 (0) 1.0000

rs10794531 C/T (R53H) *CC* *CT* *TT*  *CC* *CT* *TT*

1405 (39.32) 1672 (46.80) 496 (13.88) 1.0000 4115 (39.38) 4809 (46.02) 1526 (14.60) 0.0477

rs10820412 T/C *TT* *TC* *CC*  *TT* *TC* *CC*

918 (25.69) 1747 (48.90) 908 (25.41) 0.1919 2676 (25.61) 5186 (49.63) 2588 (24.76) 0.4572

rs2306928 A/G *AA* *AG* *GG* *AA* *AG* *GG*

3552 (99.41) 20 (0.56) 1 (0.03) 0.0319 10354 (99.08) 96 (0.92) 0 (0) 1.0000

rs849084 T/C *TT* *TC* *CC*  *TT* *TC* *CC*

976 (27.32) 1768 (49.48) 829 (23.20) 0.6151 2822 (27.01) 5175 (49.52) 2453 (23.47) 0.3996

rs12906938 T/C *TT* *TC* *CC*  *TT* *TC* *CC*

1561 (43.69) 1577 (44.14) 435 (12.17) 0.2346 4594 (43.97) 4655 (44.55) 1200 (11.48) 0.6938

rs668853 A/G *AA* *AG* *GG* *AA* *AG* *GG*

1513 (42.36) 1597 (44.71) 462 (12.93) 0.2129 4410 (42.20) 4708 (45.06) 1331 (12.74) 0.1771

rs61907183 C/T (R122C) *CC* *CT* *TT*  *CC* *CT* *TT*

3529 (98.77) 44 (1.23) 0 (0) 1.0000 10323 (98.79) 124 (1.19) 2 (0.02) 0.0576

rs139012426 G/C (S1242T) *GG* *GC* *CC*  *GG* *GC* *CC*

3464 (99.80) 7 (0.20) 0 (0) 1.0000 10177 (99.00) 103 (1.00) 0 (0) 1.0000

rs7807771 A/G *AA* *AG* *GG* *AA* *AG* *GG*

3246 (90.87) 319 (8.93) 7 (0.20) 1.0000 9425 (90.22) 993 (9.50) 29 (0.28) 0.6077

rs12068912 T/C *TT* *TC* *CC*  *TT* *TC* *CC*

2970 (83.15) 576 (16.12) 26 (0.73) 0.8346 8817 (84.38) 1561 (14.94) 71 (0.68) 0.8445

rs147317864 C/T (A262T) *CC* *CT* *TT*  *CC* *CT* *TT*

2882 (99.65) 10 (0.35) 0 (0) 1.0000 8974 (100.00) 0 (0) 0 (0) ND

rs4246302 A/G *AA* *AG* *GG* *AA* *AG* *GG*

1695 (47.47) 1567 (43.88) 309 (8.65) 0.0488 5038 (48.22) 4468 (42.77) 941 (9.01) 0.2771

____________________________________________________________________________________________________________________

Data are numbers of subjects (percentages). H-W *P*, *P* value for Hardy-Weinberg equilibrium; ND, not determined.

**Supplementary Table 3.** **Relation of SNPs to type 2 DM as determined by multivariable logistic regression analysis**

__________________________________________________________________________________________________________________________________

Dominant Recessive Additive 1 Additive 2

____________________ ____________________ ____________________ ____________________

SNP *P* OR (95% CI) *P* OR (95% CI) *P* OR (95% CI) *P* OR (95% CI)

__________________________________________________________________________________________________________________________________

rs150481868 A/G (N426S) 0.3829 0.2898 0.4478 0.2882

rs2344828 G/A (R697Q) 0.1086 0.2941 0.0566 0.3611

rs145947849 G/A (R414Q) 0.0387 0.39 (1.23–0.96) ND 0.0387 0.39 (1.23–0.96) ND

rs200803813 C/A (P176H) 0.9019 ND 0.9019 ND

rs2959953 C/G (P213R) 0.8375 0.7663 0.9113 0.7569

rs150994390 C/T (R160Q) 0.2445 0.2178 0.2760 0.2171

rs149918157 A/G (H370R) 0.6967 0.2087 0.7869 0.2084

rs2228410 C/T (D28N) 0.8681 0.6220 0.9996 0.6819

rs116429520 T/C (I114T) 0.0446 1.69 (1.01–2.76) ND 0.0446 1.69 (1.01–2.76) ND

rs190463354 G/A (R223H) 0.5806 ND 0.5806 ND

rs2070094 C/T (V488M) 0.7663 0.3539 0.9965 0.3894

rs184614603 G/A (R913Q) 0.6837 ND 0.6837 ND

rs143822500 T/C (T867A) 0.1859 0.5677 0.1942 0.5672

rs150513093 C/T (R18C) 0.7060 ND 0.7060 ND

rs117406130 T/C 0.7912 0.5667 0.8387 0.5661

rs2681417 A/G (V185I) 0.9628 ND 0.9628 ND

rs257747 C/A 0.8114 0.8658 0.8466 0.8107

rs10770 T/C (I180T) 0.7117 0.3139 0.8742 0.3110

rs201142342 T/C (I203V) 0.9819 0.3988 0.9654 0.3988

rs1053966 C/G (H1751D) 0.6940 0.5590 0.8051 0.5422

rs20571 G/A (V31I) 0.2174 0.0239 0.88 (0.79–0.98) 0.6042 0.0226 0.87 (0.77–0.98)

rs4454801 A/G 0.6959 0.3818 0.7955 0.3791

rs200312105 C/T (V995M) 0.3170 ND 0.3170 ND

rs8018102 G/A 0.5562 0.8768 0.5695 0.7534

rs75206030 C/G (P981R) 0.2882 0.2726 0.3565 0.2724

rs1419138 C/T 0.7359 0.1525 0.8855 0.2277

rs760644 A/G (T906A) 0.0812 0.3107 0.1165 0.2796

rs200981144 C/T (E211K) 0.5552 0.1089 0.6992 0.1088

rs29234 T/G 0.8940 0.4980 0.8299 0.4982

rs150534954 C/G (C155W) 0.5794 0.1353 0.4939 0.1357

rs3130977 T/C 0.9231 0.1736 0.7458 0.2050

rs12614237 T/G 0.3398 0.0853 0.6731 0.0801

rs78245253 G/C (A250P) 0.2459 0.7558 0.2209 0.7704

rs144825021 G/T (A723D) 0.9381 0.0788 0.8059 0.0789

rs144957541 G/A (S587F) 0.6778 ND 0.6778 ND

rs1353776 G/C (E729D) 0.4556 0.0258 2.15 (1.10–4.14) 0.7157 0.0254 2.16 (1.10–4.15)

rs139476663 T/C (V87A) 0.4316 ND 0.4316 ND

rs191400641 T/A (I762N) 0.6080 0.1487 0.5356 0.1490

rs2172257 T/C (T385A) 0.8806 0.7307 0.7890 0.7876

rs11177192 T/C 0.2509 0.8516 0.2002 0.6375

rs7081678 G/A 0.6099 0.1772 0.5140 0.1784

rs12151009 T/C (M180V) 0.3061 0.6852 0.3355 0.6571

rs1078485 A/G 0.5363 0.5083 0.6749 0.4338

rs13894 G/A (R126C) 0.9827 0.5031 0.8809 0.5057

rs4702982 C/T 0.8592 0.0132 1.23 (1.04–1.43) 0.5598 0.0207 1.21 (1.03–1.42)

rs11645831 G/A 0.5628 0.8594 0.5816 0.6810

rs3748045 C/G 0.2767 0.1759 0.4828 0.1346

rs11977526 A/G 0.8452 0.2640 0.8986 0.2793

rs509749 G/A (V512M) 0.1633 0.4720 0.0948 0.7174

rs138313632 T/G (S705A) **4.20 × 10^–8^** 0.20 (0.09–0.39) ND **4.20 × 10^–8^** 0.20 (0.09–0.39) ND

rs2155378 G/A 0.0452 1.09 (1.00–1.18) 0.8763 0.0390 1.10 (1.00–1.20) 0.2808

rs138559558 G/A (R289C) 0.5700 0.2742 0.6153 0.2738

rs144396411 G/A (A89T) 0.2664 0.6005 0.2733 0.5999

rs947211 G/A 0.7335 0.6157 0.8593 0.6062

rs55675869 C/T (V33366I) 0.9780 0.7261 0.9280 0.7278

rs7004867 C/T (R31Q) 0.2227 ND 0.2227 ND

rs76974938 C/T (D67N) **1.00 × 10^–23^** 0.33 (0.26–0.41) ND **1.00 × 10^–23^** 0.33 (0.26–0.41) ND

rs967417 T/C 0.9205 0.7138 0.9878 0.7137

rs2241698 G/A (V263I) 0.2477 0.4983 0.1898 0.5267

rs1992660 G/A 0.3547 0.6137 0.4092 0.5662

rs892666 T/C 0.2721 0.3883 0.1588 0.5740

rs78127944 C/T (R661H) 0.4677 0.5126 0.4257 0.5147

rs41453444 A/T (H612L) 0.7760 0.0196 6.51 (1.35–46.63) 0.9435 0.0197 6.51 (1.35–46.62)

rs34378208 C/T (G122S) 0.2129 0.4039 0.2465 0.4037

rs2228541 C/A (A246S) 0.0879 0.2852 0.1323 0.2413

rs9268507 G/A 0.6892 0.6949 0.5854 0.8199

rs72553867 C/A (T94K) 0.0303 1.10 (1.01–1.20) 0.8472 0.0288 1.11 (1.01–1.21) 0.6906

rs61731939 G/A (T833M) 0.3239 0.8844 0.3278 0.8444

rs6536541 T/C 0.0128 1.12 (1.02–1.22) 0.1383 0.0288 1.11 (1.01–1.21) 0.0935

rs7919322 G/A 0.6708 0.5827 0.7966 0.5439

rs191343457 C/G (P202R) 0.5273 ND 0.5273 ND

rs10741838 T/C 0.9898 0.7555 0.9021 0.8422

rs2351524 C/T 0.0104 1.50 (1.10–2.02) 0.2717 0.0080 1.52 (1.12–2.05) 0.2731

rs141856398 T/C (Y300C) 0.9066 0.5783 0.9231 0.5782

rs35051116 G/A (R153H) 0.7224 ND 0.7224 ND

rs10794531 C/T (R53H) 0.9648 0.1776 0.6049 0.2937

rs10820412 T/C 0.7505 0.3844 0.9809 0.4660

rs2306928 A/G 0.2116 0.0857 0.1491 0.0859

rs849084 T/C 0.2342 0.4932 0.3083 0.2573

rs12906938 T/C 0.4472 0.0972 0.8118 0.0997

rs668853 A/G 0.9719 0.7897 0.9557 0.8172

rs61907183 C/T (R122C) 0.5735 0.1767 0.4833 0.1770

rs139012426 G/C (S1242T) **2.28 × 10^–6^** 0.22 (0.09–0.44) ND **2.28 × 10^–6^** 0.22 (0.09–0.44) ND

rs7807771 A/G 0.4363 0.5705 0.4833 0.5632

rs12068912 T/C 0.0533 0.6496 0.0600 0.6025

rs147317864 C/T (A262T) **2.25 × 10^–5^** 1.21 × 10^8^ (ND) ND **2.25 × 10^–5^** 1.21 × 10^8^ (ND) ND

rs4246302 A/G 0.1989 0.4702 0.1154 0.7892

__________________________________________________________________________________________________________________________________

Multivariable logistic regression analysis was performed with adjustment for age and sex. Based on Bonferroni’s correction, *P* values of <1.44 × 10^–4^ (0.05/348) were considered statistically significant and are shown in bold. ND, not determined.

**Supplementary Table 4.** **Relation of genes, chromosomal loci, and SNPs identified in the present study to phenotypes previously examined in GWASs**

_______________________________________________________________________________________________________________

Gene (chr. SNP Nucleotide Previously examined phenotypes

locus) (amino acid)

substitution

_______________________________________________________________________________________________________________

Associated with FPG level and blood HbA_1c_ content

*CAT* rs139421991 G/A (R320Q) [Cataracts in type 2 diabetes](http://www.ebi.ac.uk/gwas/search?query=Cataracts%20in%20type%202%20diabetes) (PMID: 23137000)

*PDCL2* rs189305583 C/T (V69I) None

Associated with FPG level and type 2 DM

*RUFY1* rs138313632 T/G (S705A) [Eating disorders](http://www.ebi.ac.uk/gwas/search?query=Eating%20disorders) (PMID: 22911880)

*LOC100505549* rs139012426 G/C (S1242T) [Liver enzyme (gamma-glutamyl transferase)](http://www.ebi.ac.uk/gwas/search?query=Liver%20enzyme%20levels%20(gamma-glutamyl%20transferase)) levels (PMID: 22001757)

Associated with blood HbA_1c_ content and type 2 DM

*C21orf59* rs76974938 C/T (D67N) None

Associated with FPG level

*OR4F6* rs141569282 G/A (A117T) None

*SLC35F3* rs140011243 T/G (C144G) [Postbronchodilator FEV1/FVC ratio](http://www.ebi.ac.uk/gwas/search?query=Post%20bronchodilator%20FEV1/FVC%20ratio) (PMID: 26634245)

*KARS* rs201151665 A/T (M29L) [Pancreatic cancer](http://www.ebi.ac.uk/gwas/search?query=Pancreatic%20cancer) (PMID: 25086665)

*IFITM5* rs146230729 G/T (P31T) None

*CADM1* rs561567580 G/C (R7P) [Body mass index](http://www.ebi.ac.uk/gwas/search?query=Body%20mass%20index) (PMID: 25673413), [menarche](http://www.ebi.ac.uk/gwas/search?query=Menarche%20(age%20at%20onset)) (PMID: 25231870), [response to cytosine arabinoside](http://www.ebi.ac.uk/gwas/search?query=Response%20to%20cytadine%20analogues%20(cytosine%20arabinoside)) (PMID: 24483146), [obesity-related traits](http://www.ebi.ac.uk/gwas/search?query=Obesity-related%20traits) (PMID: 23251661), [orofacial clefts](http://www.ebi.ac.uk/gwas/search?query=Orofacial%20clefts) (PMID: 22863734)

*PPP1R9B* rs113281588 G/C (G311A) None

*MUC17* rs78010183 A/T (T1305S) None

*CCDC166* rs75368383 T/C (K187R) None

*SCAMP4* rs150715312 A/G (K69E) [Body mass index](http://www.ebi.ac.uk/gwas/search?query=Body%20mass%20index) (PMID: 25673413)

*PLEC* rs201654895 A/G (M3874V) [Postbronchodilator FEV1](http://www.ebi.ac.uk/gwas/search?query=Post%20bronchodilator%20FEV1) (PMID: 26634245), [fibrinogen levels](http://www.ebi.ac.uk/gwas/search?query=Fibrinogen%20levels) (PMID: 26561523), [body height](http://www.ebi.ac.uk/gwas/search?query=Height) (PMID: 25282103), [total cholesterol](http://www.ebi.ac.uk/gwas/search?query=Cholesterol,%20total) and [LDL-cholesterol](http://www.ebi.ac.uk/gwas/search?query=LDL%20cholesterol) (PMID: 24097068)

*LGR5* rs117324318 G/A [**Type 2 diabetes**](http://www.ebi.ac.uk/gwas/search?query=Type%202%20diabetes) (PMID: 20581827, PMID: 18372903)

*CCDC114* rs140189114 C/T (G632R) None

*NLRC3* rs116433328 G/C (M286I) [Body mass index](http://www.ebi.ac.uk/gwas/search?query=Body%20mass%20index) (PMID: 25673413)

*CECR2* rs201989565 G/A None

*DUS2* rs202069030 G/C (R51S) None

*SIGLEC1* rs201950990 A/C (V69G) None

*ALKBH1* rs200168197 A/C (V329G) [Response to platinum-based chemotherapy in non–small cell lung cancer](http://www.ebi.ac.uk/gwas/search?query=Response%20to%20platinum-based%20chemotherapy%20in%20non-small-cell%20lung%20cancer) (PMID: 22872573)

*TNFRSF4* rs150516264 A/G (L98P) [Inflammatory bowel disease](http://www.ebi.ac.uk/gwas/search?query=Inflammatory%20bowel%20disease) (PMID: 23128233)

*ADAD2* rs149894736 C/T (P107L) [Visceral adipose tissue adjusted for body mass index](http://www.ebi.ac.uk/gwas/search?query=Visceral%20adipose%20tissue%20adjusted%20for%20BMI) (PMID: 22589738)

*YBEY* rs200145138 C/G (L148V) [3-Hydroxy-1-methylpropylmercapturic acid levels in smokers](http://www.ebi.ac.uk/gwas/search?query=3-hydroxy-1-methylpropylmercapturic%20acid%20levels%20in%20smokers) (PMID: 26053186)

*PRKCDBP* rs11544766 C/G (T68S) [Diisocyanate-induced asthma](http://www.ebi.ac.uk/gwas/search?query=Diisocyanate-induced%20asthma) (PMID: 25918132), [smooth-surface caries](http://www.ebi.ac.uk/gwas/search?query=Smooth-surface%20caries) (PMID: 24556642)

*MYLIP* rs201021082 T/C (V17A) [LDL-cholesterol](http://www.ebi.ac.uk/gwas/search?query=LDL%20cholesterol) (PMID: 25961943), [diisocyanate-induced asthma](http://www.ebi.ac.uk/gwas/search?query=Diisocyanate-induced%20asthma) (PMID: 25918132), [total cholesterol](http://www.ebi.ac.uk/gwas/search?query=Cholesterol,%20total) and [LDL-cholesterol](http://www.ebi.ac.uk/gwas/search?query=LDL%20cholesterol) (PMID: 24097068), [major depressive disorder](http://www.ebi.ac.uk/gwas/search?query=Major%20depressive%20disorder) (PMID: 23377640)

*MGAT3* rs201417286 T/G (V200G) [Postbronchodilator FEV1](http://www.ebi.ac.uk/gwas/search?query=Post%20bronchodilator%20FEV1) (PMID: 26634245), [schizophrenia](http://www.ebi.ac.uk/gwas/search?query=Schizophrenia) (PMID: 26198764), [IgG glycosylation](http://www.ebi.ac.uk/gwas/search?query=IgG%20glycosylation) (PMID: 23382691)

*B3GNT6* rs559157215 A/G (H301R) None

*KNDC1* rs146093427 A/G (N546D) None

*TNXB* rs141190850 T/C (D677G) [**Type 1 diabetes and autoimmune thyroid diseases**](http://www.ebi.ac.uk/gwas/search?query=Type%201%20diabetes%20and%20autoimmune%20thyroid%20diseases) (PMID: 25936594), [systemic lupus erythematosus](http://www.ebi.ac.uk/gwas/search?query=Systemic%20lupus%20erythematosus) (PMID: 26502338, PMID: 26316170), [schizophrenia](http://www.ebi.ac.uk/gwas/search?query=Schizophrenia) (PMID: 26198764), [atopic dermatitis](http://www.ebi.ac.uk/gwas/search?query=Atopic%20dermatitis) (PMID: 25574825)

*PCDHAC1* rs185216314 A/G (Q479R) [Visceral adipose tissue adjusted for body mass index](http://www.ebi.ac.uk/gwas/search?query=Visceral%20adipose%20tissue%20adjusted%20for%20BMI) (PMID: 22589738)

*CSPG4* rs137981794 T/C (D1936G) None

*DNAJB2* rs148615702 C/G (Q235E) [Educational attainment](http://www.ebi.ac.uk/gwas/search?query=Educational%20attainment) (PMID: 25201988)

*FGD3* rs116496123 G/T None

*AK8* rs150636539 G/A (P328S) [Major depressive disorder](http://www.ebi.ac.uk/gwas/search?query=Major%20depressive%20disorder) (PMID: 23377640), [obesity-related traits](http://www.ebi.ac.uk/gwas/search?query=Obesity-related%20traits) (PMID: 23251661), [attention deficit–hyperactivity disorder](http://www.ebi.ac.uk/gwas/search?query=Attention%20deficit%20hyperactivity%20disorder) (PMID: 18839057)

*LAMB3* rs202068754 A/C (V753G) [Fentanyl consumption in laparoscopic-assisted colectomy](http://www.ebi.ac.uk/gwas/search?query=Fentanyl%20consumption%20in%20laparoscopic-assisted%20colectomy%20(first%2024%20hours)) (PMID: 26566055)

*GDPD3* rs200801803 G/A [Schizophrenia](http://www.ebi.ac.uk/gwas/search?query=Schizophrenia) (PMID: 25056061)

*OAS3* rs62623451 G/A (A49T) [Response (adiponectin levels) to fenofibrate](http://www.ebi.ac.uk/gwas/search?query=Response%20to%20fenofibrate%20(adiponectin%20levels)) (PMID: 23149075), [HDL-cholesterol](http://www.ebi.ac.uk/gwas/search?query=HDL%20cholesterol) (PMID: 21909109), [alcohol consumption](http://www.ebi.ac.uk/gwas/search?query=Alcohol%20consumption) (PMID: 21270382)

*NOTCH1* rs201053795 T/C (T970A) [Length of menstrual cycle](http://www.ebi.ac.uk/gwas/search?query=Length%20of%20menstrual%20cycle) (PMID: 26732621), [inflammatory bowel disease](http://www.ebi.ac.uk/gwas/search?query=Inflammatory%20bowel%20disease) and [Crohn’s disease](http://www.ebi.ac.uk/gwas/search?query=Crohn) (PMID: 26192919)

*SYNM* rs200549249 G/A (G235E) [Low HDL-cholesterol levels](http://www.ebi.ac.uk/gwas/search?query=Low%20high%20density%20lipoprotein%20cholesterol%20levels) (PMID: 26879886)

*SNTB1* rs145615160 A/G (Y57H) [Antipsychotic drug dosage in schizophrenia or schizoaffective disorder](http://www.ebi.ac.uk/gwas/search?query=antipsychotic%20drug%20dosage%20in%20schizophrenia%20or%20schizoaffective%20disorder) (PMID: 26821981), [periodontitis](http://www.ebi.ac.uk/gwas/search?query=Periodontitis%20(CDC/AAP)) (PMID: 24024966), [myopia](http://www.ebi.ac.uk/gwas/search?query=Myopia%20(severe)) (PMID: 23933737, PMID: 23406873), [bipolar disorder and schizophrenia](http://www.ebi.ac.uk/gwas/search?query=Bipolar%20disorder%20and%20schizophrenia) (PMID: 20889312)

Associated with blood HbA_1c_ content

*PTCHD3* rs77473776 T/G (Q186K) [**Fasting insulin–related traits**](http://www.ebi.ac.uk/gwas/search?query=Fasting%20insulin-related%20traits%20(interaction%20with%20BMI)) (PMID: 22581228), [asthma](http://www.ebi.ac.uk/gwas/search?query=Asthma%20(childhood%20onset)) (PMID: 27142222), [epilepsy and lamotrigine-induced maculopapular eruptions](http://www.ebi.ac.uk/gwas/search?query=Epilepsy%20and%20lamotrigine-induced%20maculopapular%20eruptions) (PMID: 26220383)

*TNC* rs138406927 C/T (A1096T) [Response to bupropion and depression](http://www.ebi.ac.uk/gwas/search?query=Response%20to%20bupropion%20and%20depression) (PMID: 27622933), [developmental language disorder](http://www.ebi.ac.uk/gwas/search?query=Developmental%20language%20disorder%20(syntactic%20complexity)) (PMID: 27016271), [postbronchodilator FEV1 in chronic obstructive pulmonary disease](http://www.ebi.ac.uk/gwas/search?query=Post%20bronchodilator%20FEV1%20in%20COPD) (PMID: 26634245), [plasma omega-3 polyunsaturated fatty acid level](http://www.ebi.ac.uk/gwas/search?query=Plasma%20omega-3%20polyunsaturated%20fatty%20acid%20level%20(eicosapentaenoic%20acid)) (PMID: 26584805), [cerebral amyloid deposition in APOEε4 noncarriers](http://www.ebi.ac.uk/gwas/search?query=Cerebral%20amyloid%20deposition%20in%20APOEe4%20non-carriers%20(PET%20imaging)) (PMID: 26252872)

*KRR1* rs17115182 G/A (P43S) [Polycystic ovary syndrome](http://www.ebi.ac.uk/gwas/search?query=Polycystic%20ovary%20syndrome) (PMID: 26416764), [blood Cu levels](http://www.ebi.ac.uk/gwas/search?query=Blood%20trace%20element%20(Cu%20levels)) (PMID: 23720494)

*ZNF43* rs149604219 G/A (A93V) None

*PRCP* rs2229437 T/G (E133D) [Colorectal or endometrial cancer](http://www.ebi.ac.uk/gwas/search?query=Colorectal%20or%20endometrial%20cancer) (PMID: 26621817), [urate levels](http://www.ebi.ac.uk/gwas/search?query=Urate%20levels%20(BMI%20interaction)) (PMID: 25811787)

*CYP4F12* rs609636 G/A (D76N) None

11p11.12 rs1917321 A/C Alzheimer’s disease and age of onset (PMID: 26830138), [body height](http://www.ebi.ac.uk/gwas/search?query=Height) (PMID: 20881960)

*LGALS14* rs72480733 G/A (R27H) None

*ANKRD26* rs12572862 C/G (L1219V) None

*ZKSCAN3* rs13201752 A/G (K200E) [Pulmonary function](http://www.ebi.ac.uk/gwas/search?query=Pulmonary%20function) (PMID: 21946350)

19p13.1 rs10451497 C/T None

*OR8H3* rs61751933 C/T (T16M) [Odorant perception](http://www.ebi.ac.uk/gwas/search?query=Odorant%20perception%20(β-damascenone)) (PMID: 23910658)

*HIVEP1* rs200286173 A/G (Y374C) [Oppositional defiant disorder dimensions in attention deficit–hyperactivity disorder](http://www.ebi.ac.uk/gwas/search?query=Oppositional%20defiant%20disorder%20dimensions%20in%20attention-deficit%20hyperactivity%20disorder) (PMID: 27021288), [body mass index](http://www.ebi.ac.uk/gwas/search?query=Body%20mass%20index) (PMID: 25673413), [alcohol and nicotine codependence](http://www.ebi.ac.uk/gwas/search?query=Alcohol%20and%20nictotine%20co-dependence) (PMID: 22488850), [response to antipsychotic treatment](http://www.ebi.ac.uk/gwas/search?query=Response%20to%20antipsychotic%20treatment) (PMID: 19721433)

*RSL24D1* rs200023487 A/G (V28A) [Proteinuria in chronic kidney disease](http://www.ebi.ac.uk/gwas/search?query=Proteinuria%20in%20chronic%20kidney%20disease) (PMID: 26420894), [inflammatory skin disease](http://www.ebi.ac.uk/gwas/search?query=Inflammatory%20skin%20disease) (PMID: 25574825), [neutrophil count](http://www.ebi.ac.uk/gwas/search?query=Neutrophil%20count) (PMID: 21507922)

Associated with type 2 DM

*TRABD2B* rs147317864 C/T (A262T) [Obesity-related traits](http://www.ebi.ac.uk/gwas/search?query=Obesity-related%20traits) (PMID: 23251661), [white matter integrity](http://www.ebi.ac.uk/gwas/search?query=White%20matter%20integrity) (PMID: 22425255)

_______________________________________________________________________________________________________________

Data were obtained from GWAS Catalog (http://www.ebi.ac.uk/gwas), and phenotypes related to diabetes mellitus are shown in bold. PMID, PubMed ID; chr., chromosome.

**Supplementary Table 5.** **Minor allele frequencies and effect sizes of the 56 SNPs identified in the present study**

| Gene (chr. locus) | SNP | Nucleotide (amino acid) substitution | Minor allele frequency (%) | Differences in FPG level between genotypes (%) | Differences in blood HbA_1c_ content between genotypes (%) |
| --- | --- | --- | --- | --- | --- |
| Associated with FPG level and blood HbA_1c_ content | | | | | |
| *CAT* | rs139421991 | G/A (R320Q) | 0.3 | 17.0 | 11.3 |
| *PDCL2* | rs189305583 | C/T (V69I) | 0.1 | 24.8 | 15.7 |
| Associated with FPG level and type 2 DM | | | | | |
| *RUFY1* | rs138313632 | T/G (S705A) | 0.5 | 21.3 | 22.4 |
| *LOC100505549* | rs139012426 | G/C (S1242T) | 0.4 | 20.5 | 28.0 |
| Associated with blood HbA_1c_ content and type 2 DM | | | | | |
| *C21orf59* | rs76974938 | C/T (D67N) | 2.4 | 4.3 | 9.4 |
| Associated with FPG level | | | | | |
| *OR4F6* | rs141569282 | G/A (A117T) | 1.7 | 13.8 |  |
| *SLC35F3* | rs140011243 | T/G (C144G) | 0.5 | 22.0 |  |
| *KARS* | rs201151665 | A/T (M29L) | 0.4 | 22.9 |  |
| *IFITM5* | rs146230729 | G/T (P31T) | 0.5 | 20.2 |  |
| *CADM1* | rs561567580 | G/C (R7P) | 0.4 | 20.5 |  |
| *PPP1R9B* | rs113281588 | G/C (G311A) | 0.5 | 9.6 |  |
| *MUC17* | rs78010183 | A/T (T1305S) | 1.8 | 9.9 |  |
| *CCDC166* | rs75368383 | T/C (K187R) | 0.5 | 19.7 |  |
| *SCAMP4* | rs150715312 | A/G (K69E) | 0.6 | 17.6 |  |
| *PLEC* | rs201654895 | A/G (M3874V) | 0.4 | 20.5 |  |
| *LGR5* | rs117324318 | G/A | 0.4 | 19.7 |  |
| *CCDC114* | rs140189114 | C/T (G632R) | 0.4 | 20.5 |  |
| *NLRC3* | rs116433328 | G/C (M286I) | 0.4 | 21.0 |  |
| *CECR2* | rs201989565 | G/A | 0.4 | 19.9 |  |
| *DUS2* | rs202069030 | G/C (R51S) | 0.4 | 21.3 |  |
| *SIGLEC1* | rs201950990 | A/C (V69G) | 0.5 | 18.6 |  |
| *ALKBH1* | rs200168197 | A/C (V329G) | 0.4 | 20.4 |  |
| *TNFRSF4* | rs150516264 | A/G (L98P) | 0.4 | 19.4 |  |
| *ADAD2* | rs149894736 | C/T (P107L) | 0.7 | 15.9 |  |
| *YBEY* | rs200145138 | C/G (L148V) | 0.4 | 20.2 |  |
| *PRKCDBP* | rs11544766 | C/G (T68S) | 0.4 | 19.7 |  |
| *MYLIP* | rs201021082 | T/C (V17A) | 0.4 | 20.2 |  |
| *MGAT3* | rs201417286 | T/G (V200G) | 0.3 | 20.2 |  |
| *B3GNT6* | rs559157215 | A/G (H301R) | 0.3 | 21.5 |  |
| *KNDC1* | rs146093427 | A/G (N546D) | 0.4 | 18.4 |  |
| *TNXB* | rs141190850 | T/C (D677G) | 0.3 | 21.3 |  |
| *PCDHAC1* | rs185216314 | A/G (Q479R) | 0.1 | 25.9 |  |
| *CSPG4* | rs137981794 | T/C (D1936G) | 0.4 | 18.9 |  |
| *DNAJB2* | rs148615702 | C/G (Q235E) | 0.3 | 22.1 |  |
| *FGD3* | rs116496123 | G/T | 0.7 | 14.1 |  |
| *AK8* | rs150636539 | G/A (P328S) | 0.3 | 21.0 |  |
| *LAMB3* | rs202068754 | A/C (V753G) | 0.4 | 18.3 |  |
| *GDPD3* | rs200801803 | G/A | 0.3 | 21.8 |  |
| *OAS3* | rs62623451 | G/A (A49T) | 0.3 | 19.4 |  |
| *NOTCH1* | rs201053795 | T/C (T970A) | 0.6 | 15.5 |  |
| *SYNM* | rs200549249 | G/A (G235E) | 0.2 | 23.6 |  |
| *SNTB1* | rs145615160 | A/G (Y57H) | 0.5 | 15.7 |  |
| Associated with blood HbA_1c_ content | | | | | |
| *PTCHD3* | rs77473776 | T/G (Q186K) | 30.6 |  | 9.3 |
| *TNC* | rs138406927 | C/T (A1096T) | 2.1 |  | 9.9 |
| *KRR1* | rs17115182 | G/A (P43S) | 7.0 |  | 9.3 |
| *ZNF43* | rs149604219 | G/A (A93V) | 3.0 |  | 8.6 |
| *PRCP* | rs2229437 | T/G (E133D) | 6.4 |  | 5.9 |
| *CYP4F12* | rs609636 | G/A (D76N) | 2.3 |  | 11.6 |
| 11p11.12 | rs1917321 | A/C | 35.5 |  | 3.8 |
| *LGALS14* | rs72480733 | G/A (R27H) | 36.7 |  | 3.9 |
| *ANKRD26* | rs12572862 | C/G (L1219V) | 21.7 |  | 4.2 |
| *ZKSCAN3* | rs13201752 | A/G (K200E) | 36.5 |  | 3.0 |
| 19p13.1 | rs10451497 | C/T | 31.2 |  | 4.0 |
| *OR8H3* | rs61751933 | C/T (T16M) | 18.3 |  | 2.6 |
| *HIVEP1* | rs200286173 | A/G (Y374C) | 0.2 |  | 15.6 |
| *RSL24D1* | rs200023487 | A/G (V28A) | 0.1 |  | 16.2 |
